# Discovery of Viral Myosin Genes With Complex Evolutionary History Within Plankton

**DOI:** 10.3389/fmicb.2021.683294

**Published:** 2021-06-07

**Authors:** Soichiro Kijima, Tom O. Delmont, Urara Miyazaki, Morgan Gaia, Hisashi Endo, Hiroyuki Ogata

**Affiliations:** ^1^Chemical Life Science, Institute for Chemical Research, Kyoto University, Uji, Japan; ^2^Metabolic Genomics, Genoscope, Institut de Biologie François Jacob, CEA, CNRS, Univ Evry, Université Paris Saclay, Évry-Courcouronnes, France; ^3^Laboratory of Marine Environmental Microbiology, Division of Applied Biosciences, Graduate School of Agriculture, Kyoto University, Kyoto, Japan

**Keywords:** NCLDV, giant viruses, myosin, phylogeny, viral diversity, *Nucleocytoviricota*

## Abstract

Nucleocytoplasmic large DNA viruses (NCLDVs) infect diverse eukaryotes and form a group of viruses with capsids encapsulating large genomes. Recent studies are increasingly revealing a spectacular array of functions encoded in their genomes, including genes for energy metabolisms, nutrient uptake, as well as cytoskeleton. Here, we report the discovery of genes homologous to myosins, the major eukaryotic motor proteins previously unrecognized in the virosphere, in environmental genomes of NCLDVs from the surface of the oceans. Phylogenetic analyses indicate that most viral myosins (named “virmyosins”) belong to the *Imitervirales* order, except for one belonging to the *Phycodnaviridae* family. On the one hand, the phylogenetic positions of virmyosin-encoding *Imitervirales* are scattered within the *Imitervirales*. On the other hand, *Imitervirales* virmyosin genes form a monophyletic group in the phylogeny of diverse myosin sequences. Furthermore, phylogenetic trends for the virmyosin genes and viruses containing them were incongruent. Based on these results, we argue that multiple transfers of myosin homologs have occurred not only from eukaryotes to viruses but also between viruses, supposedly during co-infections of the same host. Like other viruses that use host motor proteins for their intracellular transport or motility, these viruses may use the virally encoded myosins for the intracellular trafficking of giant viral particles.

## Introduction

Viruses were considered as tiny and simple biological objects until La Scola et al. (2003) discovered a giant virus from the water of a cooling tower. The virus named mimivirus is 750 nm in particle size and possesses a 1,182 kbp genome (Colson et al., 2017), a dimension that was large and complex enough to blow off the classical perception of viruses. After the discovery of mimivirus, related viruses were isolated including marseilleviruses, pandoraviruses, and pithoviruses, many of them with similar or even larger-sized particles or genomes (Abergel et al., 2015; Colson et al., 2017). These giant viruses infect diverse eukaryotes, possess a double-stranded DNA genome, belong to the phylum *Nucleocytoviricota* (Koonin et al., 2020), and are commonly referred to as nucleocytoplasmic large DNA viruses (NCDLVs) (Iyer et al., 2006, p. 200). The monophyletic origin of NCLDVs has been suggested based on the presence of about 40 core genes of NCLDVs that can be traced back to their putative last common ancestor (Koonin and Yutin, 2019) as well as the congruent phylogenies of the most conserved eight proteins responsible for virion morphogenesis and informational processes (Guglielmini et al., 2019).

Because of their large virions, NCLDVs can encapsulate a large genome (several hundred kb up to 2.5 Mb) in their particles. Smaller viruses (such as small RNA viruses) encode only genes that are essential for their genome replication and capsid formation, while NCLDVs encode numerous genes that are not directly involved in their genome replication and virion morphogenesis (Moniruzzaman et al., 2020). These genes, often called auxiliary metabolic genes, are considered to function in reprogramming host metabolism and molecular machinery during viral infection toward enhancing viral replication and subsequent transmission to another host. For example, the recently characterized Prymnesium kappa virus RF01 encodes genes for all four succinate dehydrogenase subunits, as well as genes for modulating β-oxidation pathway (Blanc-Mathieu et al., 2021). These viral genes are suggested to boost energy production during viral replication, which can deteriorate host metabolism, or to enhance the supply of building blocks for viral replication. Another recent study reported the presence of actin genes (viractins) in NCLDV genomes (Cunha et al., 2020). Viractins are hypothesized to help viral infections by controlling the localization of the viral factory close to the host nucleus.

Hundreds of genomes have already been sequenced for cultured NCLDVs, yet these represent only the tip of iceberg of the diverse NCLDVs uncovered through environmental surveys (Schulz et al., 2020). To bypass cultivation, genome-resolved metagenomics has been applied to large metagenomic surveys, including on oceanic samples collected by *Tara* Oceans (Sunagawa et al., 2020), in order to characterize NCLDV metagenome-assembled genomes (MAGs) containing the gene pool of thousands of those viruses (Moniruzzaman et al., 2020; Schulz et al., 2020). NCLDV MAGs revealed a cosmopolitan nature of these viruses, extensive gene transfers with eukaryotes, and their complex metabolic capabilities.

In this study, we describe the identification of myosin genes in previously published NCLDV MAGs as well as newly identified ones derived from a manual binning and curation effort focused on large cellular size fractions of *Tara* Oceans enriched in NCLDVs when infecting planktonic eukaryotes (see section “Materials and Methods”). Myosin genes, which have not been previously described in viral genomes from cultures, form a superfamily of motor proteins involved in a wide range of motility processes in eukaryotic cells. Myosins have been grouped into various classes (Odronitz and Kollmar, 2007). Most myosins are classified into class 1–35 based on their phylogenetic relationships, while other myosins are phylogenetically orphan and classified into class A to U (Odronitz and Kollmar, 2007). The functions of orphan myosins are often unknown, while the functions of some members of the class 1–35 are characterized. For example, some myosins of class 2 contract muscle (Pertici et al., 2018), while some myosins of class 5 transport specific material along actin filaments (Hammer and Sellers, 2011). The various functions of myosins are supported by the head domains (Odronitz and Kollmar, 2007), which are universally conserved among myosins, interact with actins, and frequently serve for phylogenetic analyses (Odronitz and Kollmar, 2007). The head domains of myosins interact with actins when myosins bind ADP, whereas, when ADP is absent or ATP is in the ADP-binding site of myosins, the head domains no longer interact with actins. The tail part contains various domains, including coiled-coil domains (class 2 myosins), ankyrin repeats, and IQ motifs (Odronitz and Kollmar, 2007).

## Materials and Methods

### NCLDV MAGs Derived From the Tara Ocean Project

Newly identified NCLDV MAGs were manually characterized and curated from the *Tara* Oceans metagenomes (size fractions > 0.8 μm), based on an initial binning strategy at large-scale focused on eukaryotes (Delmont et al., 2020), and following the same workflow as in previous studies (Cunha et al., 2020; Delmont et al., 2020; Kaneko et al., 2021). Briefly, metagenomes were organized into 11 sets based on their geography, and each set was co-assembled using MEGAHIT (Li et al., 2015) v.1.1.1. For each set, scaffolds longer than 2.5 kbp were processed within the bioinformatics platform anvi’o v.6.1 (Eren et al., 2015) to generate genome-resolved metagenomes (Delmont et al., 2018). CONCOCT (Alneberg et al., 2014) was used to identify large clusters of contigs within the set. We then used HMMER (Eddy, 2011) v3.1b2 to search for eight NCLDV gene markers (Guglielmini et al., 2019), and identified NCLDV MAGs by manually binning CONCOCT clusters using the anvi’o interactive interface. Finally, NCLDV MAGs were manually curated using the same interface, to minimize contamination as described previously (Delmont and Eren, 2016).

### Sequence Datasets

To prepare a sequence set for the DNA polymerase elongation subunit family B (PolB), we first extracted PolB protein sequences from NCVOG (Yutin et al., 2009). Next, we collected PolB sequences by performing BLASTP from Virus-Host DB (Mihara et al., 2016) against the PolB sequences from NCVOG. We retained hits with an E-value < 1e-10. To identify PolBs in the NCLDV MAGs, we performed BLASTP from sequences derived from NCDLV MAGs generated by Moniruzzaman et al. (2020); Schulz et al. (2020), and ourselves (*Tara* Oceans MAGs) against the PolB sequences from NCVOG and Virus-Host DB. We retained hits with an E-value < 1e-10 and with their length in a range from 800 amino acids (aa) up to 1,800 aa to exclude anomalously short or long sequences that are difficult to align. We pooled these PolB sequences, and then removed redundancy using cd-hit (4.8.1) (Li and Godzik, 2006) and manually curated the dataset to reduce its size.

To identify eight core genes of NCLDVs [DNA polymerase elongation subunit family B, D5-like helicase-primase, Poxvirus Late Transcription Factor VLTF3 like, Transcription factor S-II (TFIIS), packaging ATPase, NCLDV major capsid protein, DNA-directed RNA polymerase subunit alpha, and DNA-directed RNA polymerase subunit beta] (Koonin and Yutin, 2019) in NCLDV MAGs, we performed BLASTP (E-value < 1e-5, no length cutoff) from protein sequences derived from the MAGs against eight core genes recorded in NCVOG (Yutin et al., 2009).

For myosin homologs, we used full-length myosin sequences from a previous study (Odronitz and Kollmar, 2007) as primal references for myosins. This dataset contains various classes of myosins from diverse organisms. Next, we extracted myosin homologs from MMETSP (Keeling et al., 2014) and RefSeq (O’Leary et al., 2016) by performing BLASTP (blast 2.11.0) against the primal references to generate secondary references for full-length myosin sequences. We considered hits with an E-value < 1e-10 in this search. We identified myosin head domains in the primal and secondary myosin reference sequences by performing hmmscan (HMMER 3.3.1) (Eddy, 2011). We used Pfam (El-Gebali et al., 2019) as the HMM model for the hmmscan search and considered hits that were annotated as “Myosin_head” and showed an E-value < 1e-10. From each taxonomic group (the rank just below “Eukaryota” in the NCBI taxonomy, mostly corresponding to a class or higher level) and for each represented myosin class, we retained one representative sequence from primary and secondary reference sequences. Thus, the selected non-redundant sequence set represents myosins from wide classes and wide taxonomic ranges. To identify myosin sequences in the NCLDV MAGs, we performed BLASTP from sequences derived from MAGs against the primal myosin references. We considered hits with an E-value < 1e-10. We identified myosin head domains by performing hmmscan (Eddy, 2011). We considered hits that were annotated as “Myosin_head” with an E-value < 1e-10 and length longer than 550 aa.

### Multiple Sequence Alignment

Multiple sequence alignments were generated using MAFFT (v7.471) (Katoh et al., 2019) with the L-INS-i algorithm, which is suitable for sequences that have only one alignable domain. After multiple alignment, we removed gappy sites by using trimAl (v1.4.rev15) (Capella-Gutiérrez et al., 2009) with “-gappyout” option.

For myosin sequences from viruses and eukaryotes, we generated two trimmed alignments by removing gapped columns using “-strict” option (named “strict dataset”), in addition to the “-gappyout” option (named “gappy dataset”). Finally, we removed sequences which have more than 30% gaps along the entire length of the alignment from each dataset to exclude divergent sequences.

### Phylogenetic Analysis

The phylogenetic analyses were conducted with the ML framework using IQ-TREE (1.6.12) (Minh et al., 2020). For each alignment, we used the best substitution model selected by ModelFinder (Kalyaanamoorthy et al., 2017) of IQ-TREE. The selected models are described in the figure legends. The branch support values were computed based on the non-parametric bootstrap method with 100 bootstrap replicates, as well as transfer bootstrap expectation (TBE) with BOOSTER-WEB (Lemoine et al., 2018). TBE replaces the branch presence frequency in non-parametric bootstrap proportion (i.e., the expectation of a 0/1 function) by the expectation of a nearly continuous function. TBE supports acknowledge the presence of unstable taxa (single taxa that tends to move in and out of clades) and provide an alternative view of the robustness of a tree.

In the reconstruction of the phylogenetic tree of PolBs, *Poxviridae* sequences have been shown to be difficult to confidently position and tend to reduce the global resolution of phylogenetic trees, and were therefore subsequently removed as suggested by Guglielmini et al. (2019) to enhance the resolution of the phylogenetic trees.

For myosin sequences from viruses and eukaryotes, we also built phylogenetic trees using RAxML (8.2.12) (Stamatakis, 2014) on both “gappy” and “strict” datasets. We tried four substitution models (i.e., PROTCATAUTO, PROTCATIAUTO, PROTGAMMAAUTO, and PROTGAMMAIAUTO) and selected the tree with the highest likelihood score. Therefore, we obtained four trees for these myosin sequences (i.e., gappy/IQ-TREE, strict/IQ-TREE, gappy/RAxML, and strict/RAxML).

### Visualization

The phylogenetic trees were visualized with iTOL v4 (Letunic and Bork, 2019).

### Motif Search

We performed InterProScan (5.44-79.0) (Mitchell et al., 2019) to find motifs against each protein sequence derived from the NCLDV MAGs.

## Results and Discussion

### Myosin Genes in NCLDV Genomes

**FIGURE 1:**
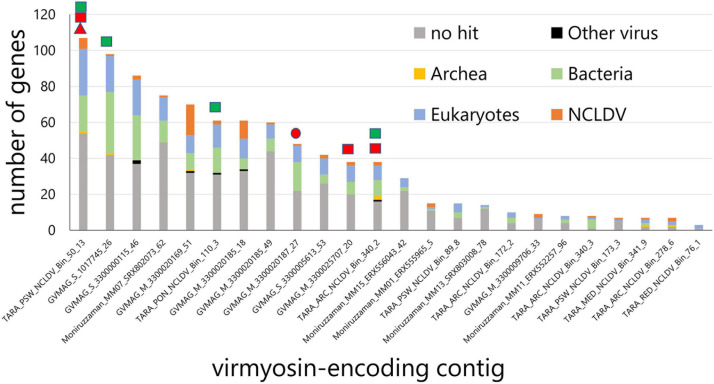
Taxonomic annotation of genes encoded in the virmyosin-harboring NCLDV contigs. Best hit-based taxonomic annotation was performed for each gene using BLAST against the RefSeq database. Identifiers on the *x*-axis represent the names of MAGs that contain virmyosin-encoding contigs. Marks above the bars indicate the presence of NCLDV core genes in the corresponding contigs, including Poxvirus Late Transcription Factor VLTF3 like (red triangle), major capsid protein (red circle), RNA polymerase subunit alpha (red rectangle), and RNA polymerase subunit beta (green rectangle).

We identified myosin-related genes in a total of 24 NCLDV MAGs (out of 2,275 considered in our survey) by performing BLASTP against reference myosin sequences compiled from a previous study (Odronitz and Kollmar, 2007), through our own independent effort from a parallel environmental genomic survey (Ha et al., 2021). These NCLDV MAGs were derived from the large cellular size fractions of *Tara* Oceans (*n* = 10) and studies by Moniruzzaman et al. (2020) (*n* = 5) and Schulz et al. (2020) (*n* = 9). All NCLDV MAGs but one originate from marine plankton samples (Supplementary Table 1). The size and number of genes in these 24 NCLDV MAGs ranged from 239 to 996 kb (633 kb on average) and 306 to 1,071 (643 on average), respectively (Supplementary Tables 1, 2). The average length of the viral myosin sequences was 964 aa (702–1,076 aa). The viral myosin sequences contain the head domain (651 aa on average, from 583 to 754 aa) and the tail domain (265 aa on average, from 5 to 381 aa). The tail domain did not show the coiled-coil domain, which is seen in the class 2 myosin (functioning in muscle). Instead, the tail domains of the virmyosins in some cases (15 out of 24) contained the IQ calmodulin-binding motif, which functions to bind calmodulin and is often seen in various classes of myosin (Supplementary Table 3) (Odronitz and Kollmar, 2007). An alignment of myosin head domain sequences from various organisms with these viral sequences establishes their strong homology (Supplementary Figure 1). Eight of 24 viral myosin genes were detected in the previously published marine metatranscriptomic data (Carradec et al., 2018) at >95% nt identify, suggesting their transcriptional activity in the ocean. The taxonomies of the closest homologs of other genes encoded together with the myosin homologs in the same contigs (Figure 1) revealed that a large proportion of genes (up to 44% and 15% on average) in the contigs best match to NCLDV genes. Some of these contigs encode NCLDV marker genes (Figure 1). Furthermore, each contig contains a large proportion of putative genes without any significant hit in public databases. Most of the viral myosin genes show a relatively low G + C content (24.2% on average), although this was slightly lower than the remaining genic regions (28.1% on average) (Supplementary Table 1). These features are specific for NCLDVs, thus excluding the possibility of contaminated myosin homologs in the NCLDV MAGs for most cases. We thus designate the viral myosin homologs as virmyosins. However, this does not mean that all the analyzed 2,275 MAGs are free of contamination from other organisms or viruses. We also recognize that the evidence for the NCLDV origin is weak for short contigs even among those shown in Figure 1.

**FIGURE 2:**
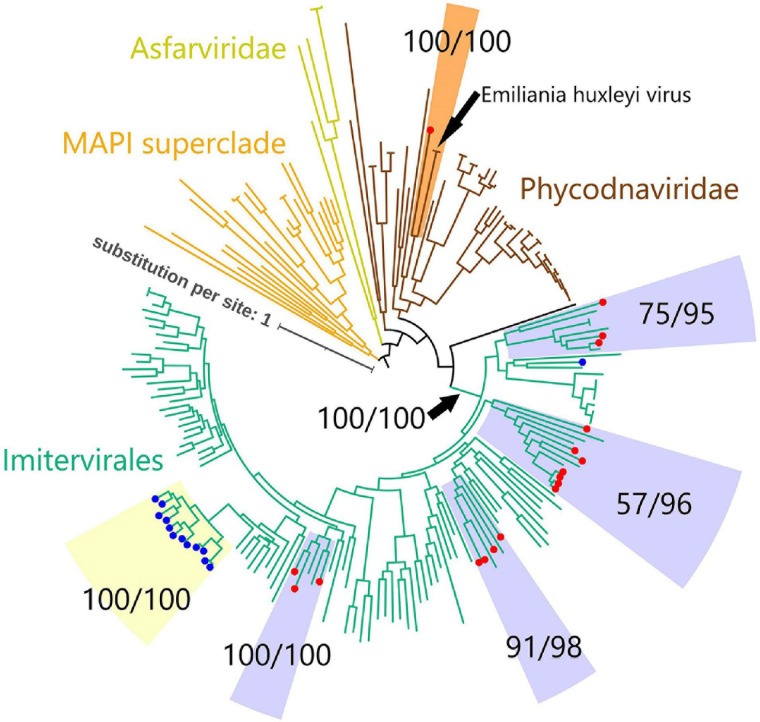
Phylogenetic tree of NCLDVs based on DNA polymerase B. The tree was built from an alignment (993 sites) of 205 PolB sequences of NCLDVs (18 MAGs encoding myosin gene, 13 MAGs encoding actin gene, and 174 other broad and unbiased taxa from the reference database). Branches labeled with red and blue circles represent NCLDV MAGs with virmyosin and viractin genes, respectively. Branches are color-coded based on family- or order-level taxonomy. The MAPI superclade (shown in orange) comprises *Marseilleviridae*, *Ascoviridae*, Pitho-like viruses, and *Iridoviridae*. Paired-numerical values represent the non-parametric bootstrap (left) and TBE (right) values for the branch support. *Phycodnaviridae* MAG with virmyosin is phylogenetically close to *Emiliania huxleyi* virus with maximum bootstrap and TBE supports. We used MAPI superclade as an outgroup to root the tree. The LG + F + R10 substitution model was selected by IQ-TREE for the best model for tree reconstruction.

To investigate the evolutionary relationships between the virmyosin-encoding NCLDVs, we performed phylogenetic analyses based on DNA polymerases (PolBs), a commonly used phylogenetic maker for NCLDVs. Eighteen of the 24 virmyosin-harboring MAGs were found to also encode PolB and were thus subjected to this analysis. The generated tree indicates that all but one of these virmyosin-encoding MAGs belong to the *Imitervirales* order (including mimiviruses and alga-infecting mimivirus relatives), the grouping of which was supported with a bootstrap value of 100% (Figure 2 and Supplementary Figure 2). However, the lineages of virmyosin-encoding MAGs were scattered within four distantly related clades in the *Imitervirales* branches (clades c1, c2, c3, and c4). Two of these clades (c3 and c4) were supported by a bootstrap value ≥ 91%, and all were supported by a TBE value ≥ 95%. The members of c1 and c3 are closely related to *Cafeteria roenbergensis* virus and *Aureococcus anophagefferens* virus, respectively. There were no closely related isolated viruses for c2 and c4. A previous study described the identification of viractin genes in two distant clades of the genomes of the *Imitervirales* order (Cunha et al., 2020). In the PolB tree we generated, the viractin-encoding MAGs were again placed within the *Imitervirales* branches but did not show close relationships with the virmyosin-encoding MAGs. One of the virmyosin-encoding MAGs was classified in the *Phycodnaviridae* family, being closely located to *Emiliania huxleyi* virus in the tree. This virmyosin was encoded in a contig harboring seven genes (MAG: TARA_ARC_NCLDV_Bin_278_6; contig: c_000000000006), of which two best hit to NCLDVs (Figure 1). Given the small size of the contig, the possibility of contamination cannot be totally excluded for this contig.

Six of the 24 virmyosin-harboring MAGs did not encode PolB. When searched against reference isolated viral genomes, most of the NCLDV core genes from these six MAGs showed the largest sequence similarities to the viruses of *Imitervirales*, suggesting that they are the members of this order (Supplementary Table 4).

**FIGURE 3:**
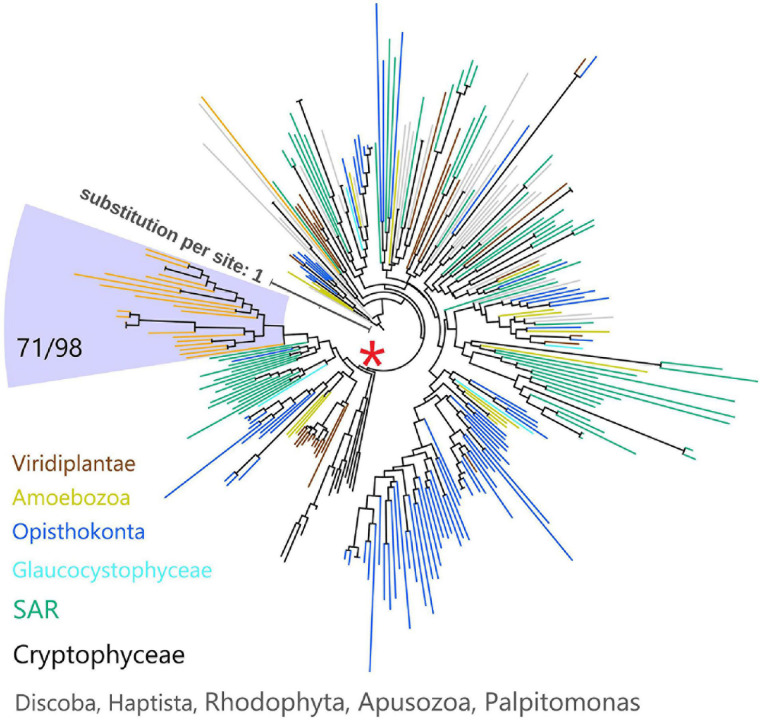
Phylogeny of myosin from NCLDVs and eukaryotic lineages. The tree was built from the multiple sequence alignment (658 sites) of 286 myosin head domain sequences. Branches are color-coded based on taxonomic groups (orange branches correspond to virmyosins). The *Imitervirales* virmyosin clade (highlighted with a pale blue-colored background) was supported with a bootstrap value of 71% of and TBE of 98%. We consider myosin-II as an outgroup to root this tree. The LG + R10 substitution model was selected by IQ-TREE for the best model for tree reconstruction.

Since myosins are universal in the eukaryotic domain, we hypothesized that these NCLDVs acquired myosin homologs by horizontal gene transfers (HGTs) from various eukaryotes. To determine the source eukaryotic lineages for the putative HGTs, we performed phylogenetic analyses on the virmyosin and reference myosin sequences. We generated four phylogenetic trees with different methods (Figure 3 and Supplementary Figures 3A–E; see section “Materials and Methods”). The phylogenetic placement of the virmyosin from the MAG belonging to the *Phycodnaviridae* family was unstable. However, all the trees showed a monophyletic group of *Imitervirales* virmyosins. The grouping is supported by a bootstrap value of 71% and TBE of 98% (Figure 3 and Supplementary Figure 3E).

**FIGURE 4:**
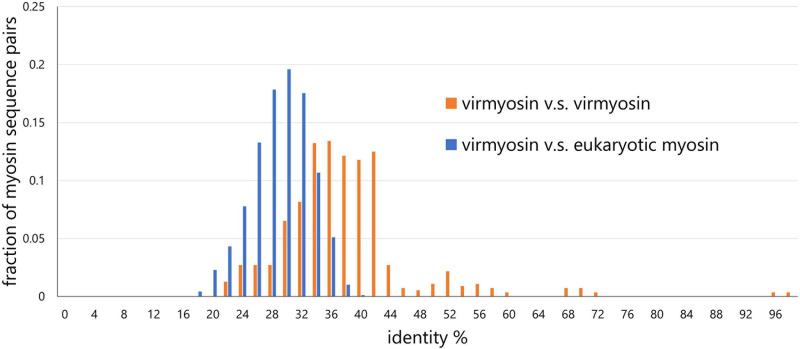
Distributions of pair-wise myosin sequence similarities within the NCLDVs (virmyosin *vs* virmyosin) and between NCLDVs and eukaryotes (virmyosin *vs* eukaryotic myosin). This graph shows that the mean value of myosin similarity within NCLDVs is higher than that between NCLDVs and eukaryotes.

**FIGURE 5:**
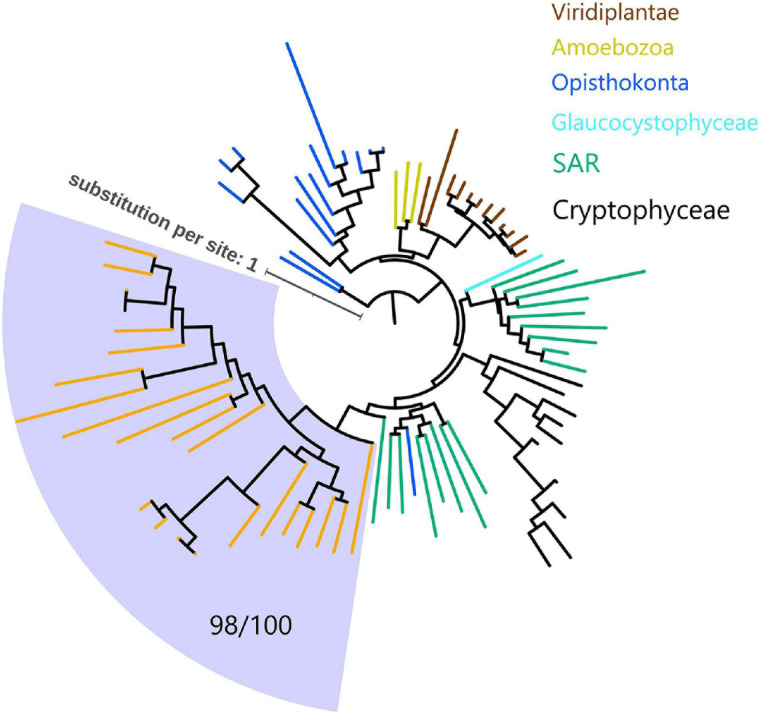
Phylogeny of virmyosins and their close relatives in eukaryotes. The tree was built from an alignment (688 sites) of 81 myosin sequences of NCLDVs, Amoebozoa, SAR (Stramenopiles, Alveolata, and Rhizaria), Opisthokonta, Cryptophyceae, Viridiplantae, and Glaucocystophyceae. Background of the *Imitervirales* virmyosin clade is pale blue-colored. Branches are color-coded based on taxonomic groups (orange branches correspond to virmyosins). We consider myosin-II of *Nasonia vitripennis* and *Neurospora crassa* as outgroups to root this tree. The LG + F + R7 substitution model was selected by IQ-TREE for the best model for tree reconstruction.

To examine the effect of long-branch attraction on the formation of the *Imitervirales* virmyosin clade, we computed the pair-wise sequence identities within virmyosins and between virmyosins and eukaryotic myosins (Figure 4). The sequence similarities for many of the virmyosin pairs were higher than those between virmyosins and eukaryotic myosins, thus diminishing the possibility of long branch attraction effect on the monophyletic grouping the *Imitervirales* virmyosins. The *Imitervirales* virmyosin clade is branching out from orphan myosins of the SAR (Stramenopiles, Alveolates, and Rhizaria) supergroup, but the grouping of these virmyosins and the orphan myosins was not supported. Finally, to further improve the tree reconstruction, we built a phylogenetic tree using the virmyosin sequences as well as eukaryotic myosin sequences that formed a clade together with the virmyosins (the clade marked with red “^∗^” in Figure 3). The newly generated tree again displayed the monophyletic grouping of the *Imitervirales* virmyosins (bootstrap value, 98% and TBE, 100%) and placed it within a clade of myosin sequences from Apicomplexa (*Toxoplasma gondii*), Stramenopiles (the diatom *Thalassiosira pseudonana*, and the oomycete *Hyaloperonospora parasitica*), and a fungus (*Rhizopogon burlinghamii*) (Figure 5 and Supplementary Figures 3F,G). We further regenerated a myosin tree by adding diverse eukaryotic myosin sequences (Supplementary Figures 3H,I). The tree showed a monophyletic group of the virmyosins within a clade enriched with sequences from SAR. However, the grouping of the virmyosins and eukaryotic sequences was not supported. This grouping and the fact that eukaryotes belonging to the SAR supergroup represent a major group of marine plankton suggest a possible SAR origin for virmyosins but leave the specific source lineage unresolved due to the lack of statistical support.

### Possible HGT Scenarios for the Virmyosin

**FIGURE 6:**
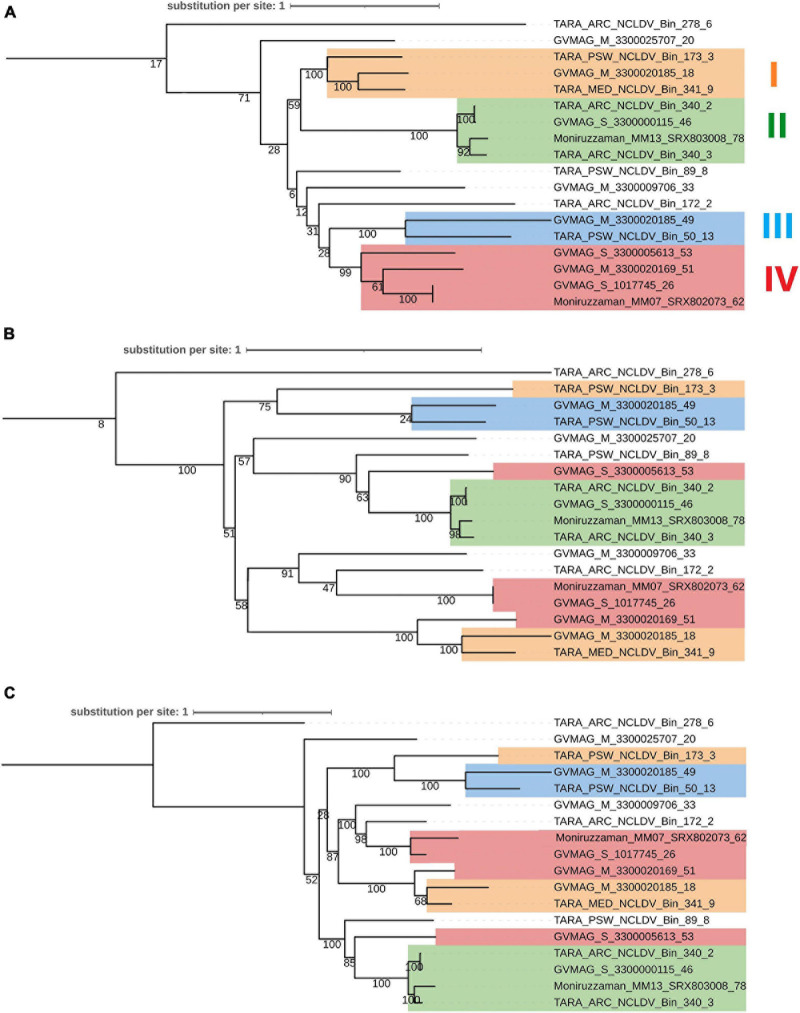
Phylogenetic trees of **(A)** viral myosins, **(B)** PolBs, and **(C)** core gene sequences from MAGs encoding both genes myosin and PolB. Labels at the leaves are the identifiers of the NCLDV MAGs. Clades supported with a parametric bootstrap value > 99% in the myosin tree are marked in both trees by colored rectangles. This myosin and PolB trees were generated from Figure 3 and Figure 2 by extracting MAGs encoding both myosin and PolB, respectively. Clades I–IV correspond to highly supported groups in the myosin tree.

We next examined the congruence of the tree of virmyosins and the viral trees based on PolB and eight core genes of NCLDVs. In this analysis, we focused on 18 MAGs that encode both virmyosin and PolB. Congruence of the topologies between these trees (the virmyosin tree and viral trees) is expected if the original virmyosin gene was acquired by an ancestral virus prior to the divergence of viral families or orders. We extracted subtrees for 18 virmyosins from Figure 3 and 18 PolBs from Figure 2 (Figures 6A,B). The tree based on the NCLDV core genes was newly reconstructed (Figure 6C), which was mostly congruent with the tree based on PolB (Figure 6B). To investigate the congruence of the trees, we focused on clades in the virmyosin tree that were supported with a bootstrap value greater than 99%. The analysis revealed the clades II and III form monophyletic groups also in the viral trees. However, the monophyletic clades I and IV in the virmyosin tree turned out to be polyphyletic in the viral trees with statistical supports (Figure 6). This result suggests that multiple and distantly related ancestral viruses of *Imitervirales* independently acquired myosin genes through HGTs.

The monophyletic grouping of the *Imitervirales* virmyosins and the scattered distribution of the virmyosin-encoding *Imitervirales* in the viral trees are intriguing. We consider four major possibilities for the origin of virmyosins. The first possibility is that all the *Imitervirales* myosin genes were vertically inherited from an ancestral virus of the *Imitervirales*, and subsequently lost independently in most of the descendant lineages. This scenario is not parsimonious as it needs to assume many independent gene losses, given 24 virmyosin-encoding NCLDVs and over 2,000 NCLDVs lacking virmyosin. The second possibility is that myosin genes were independently recruited multiple times from eukaryotes by ancestral viruses belonging to the *Imitervirales*. This scenario can account for the topological differences between the virmyosin and viral trees. However, this scenario cannot readily explain the monophyletic grouping of the virmyosins, as this scenario needs to additionally assume independent horizontal acquisitions of myosin genes by distantly related ancestral viruses from the same or closely related eukaryotes (e.g., ancestral SAR). Such acquisitions seem implausible because host changes are likely rampant events for *Imitervirales* given the wide host ranges of the known viruses in this group (Sun et al., 2020). The third possibility is that a myosin gene transfer occurred once in a viral lineage of *Imitervirales* from its host. Then, after the viral myosin gene acquired beneficial functions for the virus, this gene was transferred to other viruses, probably during co-infection in the same host (which may be different from the current hosts). Of note, a previous study reported a clear case of HGT between NCLDVs (Christo-Foroux et al., 2020). This scenario can explain both the topological difference between the virmyosin and viral trees and the monophyletic grouping of the virmyosins. Finally, as a remote possibility, myosin genes could have been transferred from an ancient giant virus to a eukaryotic ancestor [either before or after the Last Eukaryotic Common Ancestor (LECA)] as suggested for DNA-dependent RNA polymerases (Guglielmini et al., 2019). This scenario is not supported by the tree topology of myosins. However, given the unresolved deep branches in the tree especially for the virmyosin clade, this scenario cannot be excluded as a possible one. In this case, gene transfers between NCLDVs still need to be invoked to account for the monophyly of virmyosins. Further exploration of the actual diversity of *Imitervirales*, and more globally of NCLDVs, from various environments will certainly provide important insights regarding the robustness of these hypotheses.

### Possible Roles of Virmyosin

Myosins are known to walk along actin filaments, which are located at the peripheric side of the cytoplasm (Geada et al., 2001; Handa et al., 2013). For example, the actin and microtubule cytoskeletons cooperate in organelle transport in a variety of situations (Goode et al., 2000; Rogers and Gelfand, 2000). Secretory vesicles also move by both microtubule- and actin-based molecular motors (Geada et al., 2001). Some classes of myosins function as a transporter to carry specific materials including viruses. For example, in the case of human immunodeficiency virus and influenza A virus, virus-containing endocytic viral cargoes propelled by class 2 myosin moving on filamentous actin spread to neighboring uninfected cells (Kadiu and Gendelman, 2011; Roberts et al., 2015). Motor proteins are used for the intracellular motility of NCLDVs. For example, motor proteins such as kinesin and dynein are known to transport African swine fever virus on microtubules (Jouvenet and Wileman, 2005). Intracellular enveloped viruses of vaccinia virus are transported toward the cell surface in a microtubule-dependent process (Hollinshead et al., 2001). Virmyosin identified in the environmental NCLDV genomes in this study may function in intracellular transport of virions like these viruses. Of note, there is no clear case for the use of myosin molecular motors by NCLDVs for their intracellular transport or motility. It has been speculated that the slow movements of intracellular enveloped virus of vaccinia virus that persist in the absence of microtubules may be related to the action of myosin motors (Geada et al., 2001). It is also known that the formation of actin tail, which propels virions of vaccinia virus, requires host Myosin-9A (Handa et al., 2013), but the role Myosin-9A as molecular motor in this process is unknown.

## Conclusion

In this study, we provided strong evidence showing that marine members of NCLDVs encode myosin homologs (virmyosins). The function of virmyosin could not be inferred based on the similarity to functionally characterized myosin homologs. Together with the previously discovered actin homologs in NCLDVs, these results suggest that the genetic independence of NCLDVs from their hosts encompasses a wide-range of cellular processes, including intracellular trafficking as implied by our study and the translation process as considered from previous discoveries of translation genes in this group of viruses. Our phylogenetic analyses suggest a complex evolutionary origin of the virmyosin genes, which may involve not only HGTs from eukaryotes to NCLDVs but also intra-virus HGTs within NCLDVs. The functions encoded in the huge genetic pool of NCLDVs are revealing the amazingly diverse strategies to control host cellular processes in this diverse group of viruses. Scrutinizing available and newly generated environmental genomes will contribute to better characterizing the infection strategies of this fascinating group of viruses.

## Data Availability Statement

*Tara* Oceans raw read data analyzed in Delmont et al. (2020) and the present study are available at the EBI under project PRJEB402 (https://www.ebi.ac.uk/ena/browser/view/PRJEB402). All the NCLDV MAG data presented in this study, as well as associated data (e.g., sequence datasets, multiple sequence alignments, and phylogenetic trees) are available from ftp://ftp.genome.jp/pub/db/community/tara/Virmyosin/ and https://www.genome.jp/ftp/db/community/tara/Virmyosin/.

## Author Contributions

SK performed most of the bioinformatics analyses and wrote the initial version of the manuscript. TD performed metagenomic analyses. UM contributed to the discovery of viral myosin genes. MG contributed to the phylogenetic and evolutionary analyses. HO designed the project. HE and HO supervised the work. All authors contributed to the interpretation of the data and finalization of the manuscript, and approved the final manuscript.

## Conflict of Interest

The authors declare that the research was conducted in the absence of any commercial or financial relationships that could be construed as a potential conflict of interest.
