# Supplementary figures and images for: Discovery of Viral Myosin Genes With Complex Evolutionary History Within Plankton
Source: Front Microbiol. 2021 Jun 7;12:683294. doi: 10.3389/fmicb.2021.683294 (PMC8215601; doi:10.3389/fmicb.2021.683294)

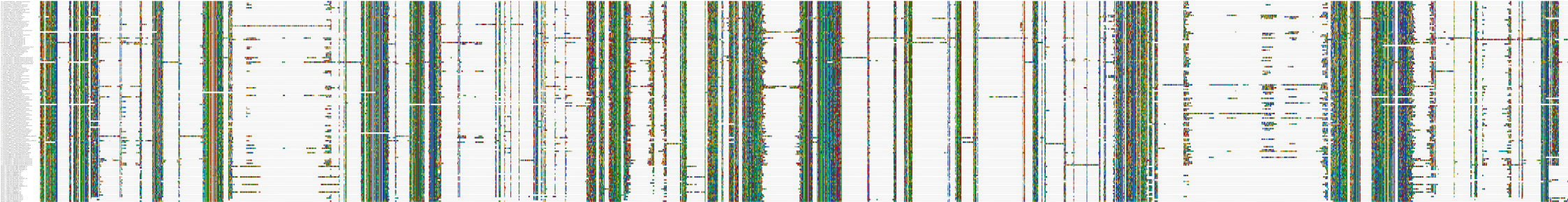

Supplement: Supplementary file 5 [file Data_Sheet_1.PDF]
