# Supplementary material for: Discovery of Viral Myosin Genes With Complex Evolutionary History Within Plankton
Source: Front Microbiol. 2021 Jun 7;12:683294. doi: 10.3389/fmicb.2021.683294 (PMC8215601; doi:10.3389/fmicb.2021.683294)
Supplement: Supplementary file 6 [file Data_Sheet_2.PDF]

Supplementary Figure 2 | Phylgenetic trees of DNA polymerase B (PolB) sequences. **(A)** Phylogenetic tree of NCLDVs based on PolB. The tree was built from an alignment (993 sites) of 205 PolB sequences of NCLDVs. Numerical values represent the non-parametric bootstrap values for the branch support. The labels of PolBs whose MAG encodes virmyosins are marked with red circle. We used MAPI superclade (Marseilleviridae, Ascoviridae, Pitho-like viruses and Iridoviridae) as an outgroup to root the tree. The LG+F+R10 substitution model was selected by IQ-TREE for the best model for tree reconstruction. **(B)** Phylogenetic tree of NCLDVs based on PolB. Numerical values represent the transfer bootstrap expectation for the branch support. The labels of PolBs whose MAG encodes virmyosins are marked with red circle. We used MAPI superclade as an outgroup to root the tree.

A

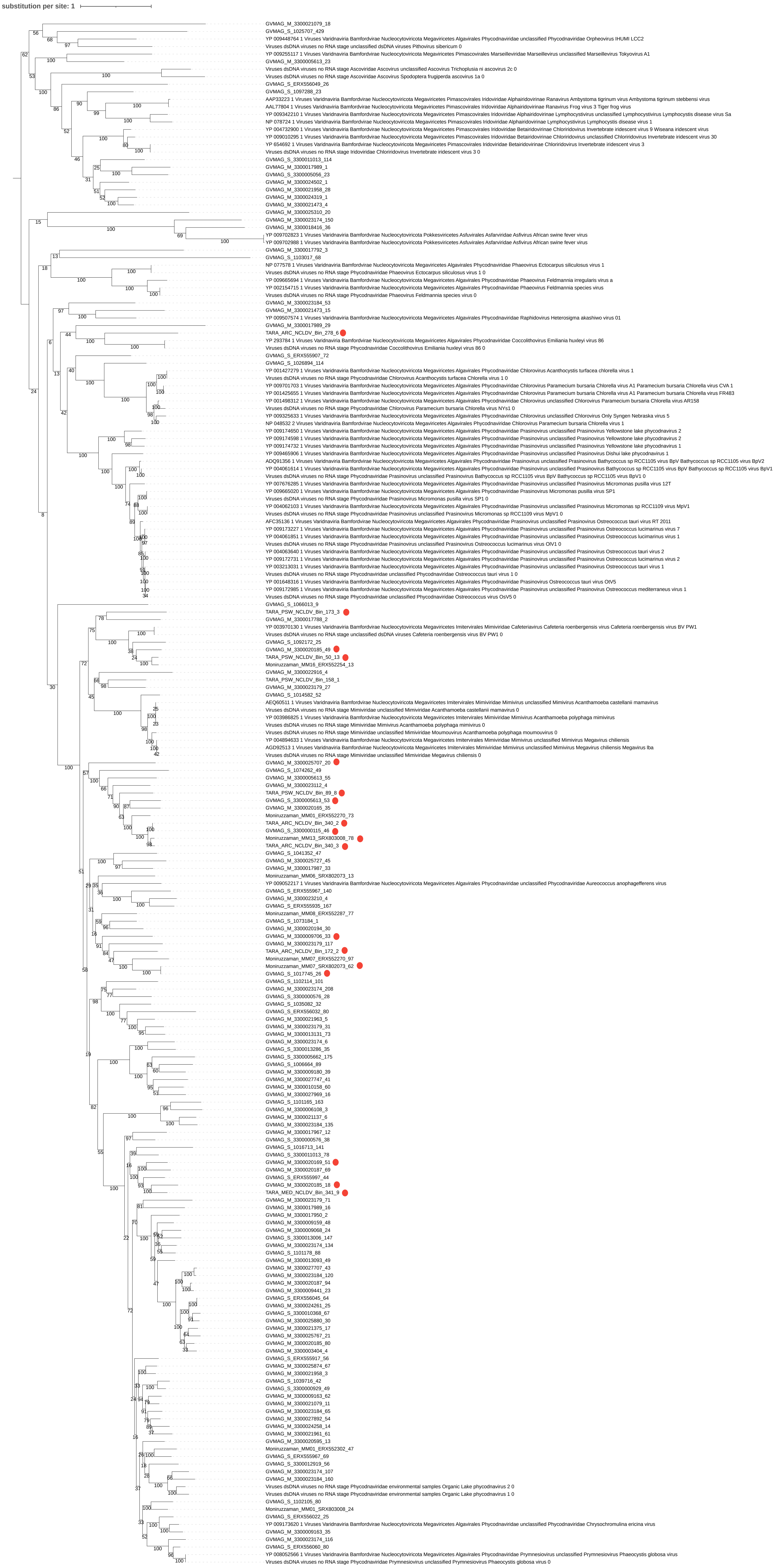
substitution per site: 1
GVMAG_M_3300021079_18
GVMAG_S_1025707_429
YP 009448764 1 Viruses Varidnaviria Bamfordvirae Nucleocytoviricota Megaviricetes Algavirales Phycodnaviridae unclassified Phycodnaviridae Orpheovirus IHUMI LCC2
Viruses dsDNA viruses no RNA stage unclassified dsDNA viruses Pithovirus sibericum 0
YP 009255117 1 Viruses Varidnaviria Bamfordvirae Nucleocytoviricota Megaviricetes Pimascovirales Marseilleviridae Marseillevirus unclassified Marseillevirus Tokyovirus A1
GVMAG_M_3300005613_23
Viruses dsDNA viruses no RNA stage Ascoviridae Ascovirus unclassified Ascovirus Trichoplusia ni ascovirus 2c 0
Viruses dsDNA viruses no RNA stage Ascoviridae Ascovirus Spodoptera frugiperda ascovirus 1a 0
GVMAG_S_ERX556049_26
GVMAG_S_1097288_23
AAP33223 1 Viruses Varidnaviria Bamfordvirae Nucleocytoviricota Megaviricetes Pimascovirales Iridoviridae Alphairidovirinae Ranavirus Ambystoma tigrinum virus Ambystoma tigrinum stebbensi virus
AAL77804 1 Viruses Varidnaviria Bamfordvirae Nucleocytoviricota Megaviricetes Pimascovirales Iridoviridae Alphairidovirinae Ranavirus Frog virus 3 Tiger frog virus
YP 009342210 1 Viruses Varidnaviria Bamfordvirae Nucleocytoviricota Megaviricetes Pimascovirales Iridoviridae Alphairidovirinae Lymphocystivirus unclassified Lymphocystivirus Lymphocystis disease virus Sa
NP 078724 1 Viruses Varidnaviria Bamfordvirae Nucleocytoviricota Megaviricetes Pimascovirales Iridoviridae Alphairidovirinae Lymphocystivirus Lymphocystis disease virus 1
YP 004732900 1 Viruses Varidnaviria Bamfordvirae Nucleocytoviricota Megaviricetes Pimascovirales Iridoviridae Betairidovirinae Chloriridovirus Invertebrate iridescent virus 9 Wiseana iridescent virus
YP 009010295 1 Viruses Varidnaviria Bamfordvirae Nucleocytoviricota Megaviricetes Pimascovirales Iridoviridae Betairidovirinae Chloriridovirus unclassified Chloriridovirus Invertebrate iridescent virus 30
YP 654692 1 Viruses Varidnaviria Bamfordvirae Nucleocytoviricota Megaviricetes Pimascovirales Iridoviridae Betairidovirinae Chloriridovirus Invertebrate iridescent virus 3
Viruses dsDNA viruses no RNA stage Iridoviridae Chloriridovirus Invertebrate iridescent virus 3 0
GVMAG_S_3300011013_114
GVMAG_M_3300017989_1
GVMAG_S_3300005056_23
GVMAG_M_3300024502_1
GVMAG_M_3300021958_28
GVMAG_M_3300024319_1
GVMAG_M_3300021473_4
GVMAG_M_3300025310_20
GVMAG_M_3300023174_150
GVMAG_M_3300018416_36
YP 009702823 1 Viruses Varidnaviria Bamfordvirae Nucleocytoviricota Pokkesviricetes Asfuvirales Asfarviridae Asfivirus African swine fever virus
YP 009702988 1 Viruses Varidnaviria Bamfordvirae Nucleocytoviricota Pokkesviricetes Asfuvirales Asfarviridae Asfivirus African swine fever virus
GVMAG_M_3300017792_3
GVMAG_S_1103017_68
NP 077578 1 Viruses Varidnaviria Bamfordvirae Nucleocytoviricota Megaviricetes Algavirales Phycodnaviridae Phaeovirus Ectocarpus siliculosus virus 1
Viruses dsDNA viruses no RNA stage Phycodnaviridae Phaeovirus Ectocarpus siliculosus virus 1 0
YP 009665694 1 Viruses Varidnaviria Bamfordvirae Nucleocytoviricota Megaviricetes Algavirales Phycodnaviridae Phaeovirus Feldmannia irregularis virus a
YP 002154715 1 Viruses Varidnaviria Bamfordvirae Nucleocytoviricota Megaviricetes Algavirales Phycodnaviridae Phaeovirus Feldmannia species virus
Viruses dsDNA viruses no RNA stage Phycodnaviridae Phaeovirus Feldmannia species virus 0
GVMAG_M_3300023184_53
GVMAG_M_3300021473_15
YP 009507574 1 Viruses Varidnaviria Bamfordvirae Nucleocytoviricota Megaviricetes Algavirales Phycodnaviridae Raphidovirus Heterosigma akashiwo virus 01
GVMAG_M_3300017989_29
TARA_ARC_NCLDV_Bin_278_6
YP 293784 1 Viruses Varidnaviria Bamfordvirae Nucleocytoviricota Megaviricetes Algavirales Phycodnaviridae Coccolithovirus Emiliania huxleyi virus 86
Viruses dsDNA viruses no RNA stage Phycodnaviridae Coccolithovirus Emiliania huxleyi virus 86 0
GVMAG_S_ERX555907_72
GVMAG_S_1026894_114
YP 001427279 1 Viruses Varidnaviria Bamfordvirae Nucleocytoviricota Megaviricetes Algavirales Phycodnaviridae Chlorovirus Acanthocystis turfacea chlorella virus 1
Viruses dsDNA viruses no RNA stage Phycodnaviridae Chlorovirus Acanthocystis turfacea Chlorella virus 1 0
YP 009701703 1 Viruses Varidnaviria Bamfordvirae Nucleocytoviricota Megaviricetes Algavirales Phycodnaviridae Chlorovirus Paramecium bursaria Chlorella virus A1 Paramecium bursaria Chlorella virus CVA 1
YP 001425655 1 Viruses Varidnaviria Bamfordvirae Nucleocytoviricota Megaviricetes Algavirales Phycodnaviridae Chlorovirus Paramecium bursaria Chlorella virus A1 Paramecium bursaria Chlorella virus FR483
YP 001498312 1 Viruses Varidnaviria Bamfordvirae Nucleocytoviricota Megaviricetes Algavirales Phycodnaviridae Chlorovirus unclassified Chlorovirus Paramecium bursaria Chlorella virus AR158
Viruses dsDNA viruses no RNA stage Phycodnaviridae Chlorovirus Paramecium bursaria Chlorella virus NYs1 0
YP 009325633 1 Viruses Varidnaviria Bamfordvirae Nucleocytoviricota Megaviricetes Algavirales Phycodnaviridae Chlorovirus unclassified Chlorovirus Only Syngen Nebraska virus 5
NP 048532 2 Viruses Varidnaviria Bamfordvirae Nucleocytoviricota Megaviricetes Algavirales Phycodnaviridae Chlorovirus Paramecium bursaria Chlorella virus 1
YP 009174650 1 Viruses Varidnaviria Bamfordvirae Nucleocytoviricota Megaviricetes Algavirales Phycodnaviridae Prasinovirus unclassified Prasinovirus Yellowstone lake phycodnavirus 2
YP 009174598 1 Viruses Varidnaviria Bamfordvirae Nucleocytoviricota Megaviricetes Algavirales Phycodnaviridae Prasinovirus unclassified Prasinovirus Yellowstone lake phycodnavirus 2
YP 009174732 1 Viruses Varidnaviria Bamfordvirae Nucleocytoviricota Megaviricetes Algavirales Phycodnaviridae Prasinovirus unclassified Prasinovirus Yellowstone lake phycodnavirus 1
YP 009465906 1 Viruses Varidnaviria Bamfordvirae Nucleocytoviricota Megaviricetes Algavirales Phycodnaviridae Prasinovirus unclassified Prasinovirus Dishui lake phycodnavirus 1
ADQ91356 1 Viruses Varidnaviria Bamfordvirae Nucleocytoviricota Megaviricetes Algavirales Phycodnaviridae Prasinovirus unclassified Prasinovirus Bathycoccus sp RCC1105 virus BpV Bathycoccus sp RCC1105 virus BpV2
YP 004061614 1 Viruses Varidnaviria Bamfordvirae Nucleocytoviricota Megaviricetes Algavirales Phycodnaviridae Prasinovirus unclassified Prasinovirus Bathycoccus sp RCC1105 virus BpV Bathycoccus sp RCC1105 virus BpV1
Viruses dsDNA viruses no RNA stage Phycodnaviridae Prasinovirus unclassified Prasinovirus Bathycoccus sp RCC1105 virus BpV Bathycoccus sp RCC1105 virus BpV1 0
YP 007676285 1 Viruses Varidnaviria Bamfordvirae Nucleocytoviricota Megaviricetes Algavirales Phycodnaviridae Prasinovirus unclassified Prasinovirus Micromonas pusilla virus 12T
YP 009665020 1 Viruses Varidnaviria Bamfordvirae Nucleocytoviricota Megaviricetes Algavirales Phycodnaviridae Prasinovirus Micromonas pusilla virus SP1
Viruses dsDNA viruses no RNA stage Phycodnaviridae Prasinovirus Micromonas pusilla virus SP1 0
YP 004062103 1 Viruses Varidnaviria Bamfordvirae Nucleocytoviricota Megaviricetes Algavirales Phycodnaviridae Prasinovirus unclassified Prasinovirus Micromonas sp RCC1109 virus MpV1
Viruses dsDNA viruses no RNA stage Phycodnaviridae Prasinovirus unclassified Prasinovirus Micromonas sp RCC1109 virus MpV1 0
AFC35136 1 Viruses Varidnaviria Bamfordvirae Nucleocytoviricota Megaviricetes Algavirales Phycodnaviridae Prasinovirus unclassified Prasinovirus Ostreococcus tauri virus RT 2011
YP 009173227 1 Viruses Varidnaviria Bamfordvirae Nucleocytoviricota Megaviricetes Algavirales Phycodnaviridae Prasinovirus unclassified Prasinovirus Ostreococcus lucimarinus virus 7
YP 004061851 1 Viruses Varidnaviria Bamfordvirae Nucleocytoviricota Megaviricetes Algavirales Phycodnaviridae Prasinovirus unclassified Prasinovirus Ostreococcus lucimarinus virus 1
Viruses dsDNA viruses no RNA stage Phycodnaviridae Prasinovirus unclassified Prasinovirus Ostreococcus lucimarinus virus OlV1 0
YP 004063640 1 Viruses Varidnaviria Bamfordvirae Nucleocytoviricota Megaviricetes Algavirales Phycodnaviridae Prasinovirus unclassified Prasinovirus Ostreococcus tauri virus 2
YP 009172731 1 Viruses Varidnaviria Bamfordvirae Nucleocytoviricota Megaviricetes Algavirales Phycodnaviridae Prasinovirus unclassified Prasinovirus Ostreococcus lucimarinus virus 2
YP 003213031 1 Viruses Varidnaviria Bamfordvirae Nucleocytoviricota Megaviricetes Algavirales Phycodnaviridae Prasinovirus unclassified Prasinovirus Ostreococcus tauri virus 1
Viruses dsDNA viruses no RNA stage Phycodnaviridae unclassified Phycodnaviridae Ostreococcus tauri virus 1 0
YP 001648316 1 Viruses Varidnaviria Bamfordvirae Nucleocytoviricota Megaviricetes Algavirales Phycodnaviridae Prasinovirus Ostreococcus tauri virus OtV5
YP 009172985 1 Viruses Varidnaviria Bamfordvirae Nucleocytoviricota Megaviricetes Algavirales Phycodnaviridae Prasinovirus unclassified Prasinovirus Ostreococcus mediterraneus virus 1
Viruses dsDNA viruses no RNA stage Phycodnaviridae unclassified Phycodnaviridae Ostreococcus virus OsV5 0
GVMAG_S_1066013_9
TARA_PSW_NCLDV_Bin_173_3
GVMAG_M_3300017788_2
YP 003970130 1 Viruses Varidnaviria Bamfordvirae Nucleocytoviricota Megaviricetes Imitervirales Mimiviridae Cafeteriavirus Cafeteria roenbergensis virus Cafeteria roenbergensis virus BV PW1
Viruses dsDNA viruses no RNA stage unclassified dsDNA viruses Cafeteria roenbergensis virus BV PW1 0
GVMAG_S_1092172_25
GVMAG_M_3300020185_49
TARA_PSW_NCLDV_Bin_50_13
Moniruzzaman_MM16_ERX552254_13
GVMAG_M_3300022916_4
TARA_PSW_NCLDV_Bin_158_1
GVMAG_M_3300023179_27
GVMAG_S_1014582_52
AEQ60511 1 Viruses Varidnaviria Bamfordvirae Nucleocytoviricota Megaviricetes Imitervirales Mimiviridae Mimivirus unclassified Mimivirus Acanthamoeba castellanii mamavirus
Viruses dsDNA viruses no RNA stage unclassified Mimiviridae Acanthamoeba castellanii mamavirus 0
YP 003986825 1 Viruses Varidnaviria Bamfordvirae Nucleocytoviricota Megaviricetes Imitervirales Mimiviridae Mimivirus Acanthamoeba polyphaga mimivirus
Viruses dsDNA viruses no RNA stage Mimiviridae Mimivirus Acanthamoeba polyphaga mimivirus 0
Viruses dsDNA viruses no RNA stage Mimiviridae unclassified Mimiviridae Moumouvirus Acanthamoeba polyphaga moumouvirus 0
YP 004894633 1 Viruses Varidnaviria Bamfordvirae Nucleocytoviricota Megaviricetes Imitervirales Mimiviridae Mimivirus unclassified Mimivirus Megavirus chiliensis
AGD92513 1 Viruses Varidnaviria Bamfordvirae Nucleocytoviricota Megaviricetes Imitervirales Mimiviridae Mimivirus unclassified Mimivirus Megavirus chiliensis Megavirus lba
Viruses dsDNA viruses no RNA stage Mimiviridae unclassified Mimiviridae Megavirus chiliensis 0
GVMAG_M_3300025707_20
GVMAG_S_1074262_49
GVMAG_M_3300005613_55
GVMAG_M_3300023112_4
TARA_PSW_NCLDV_Bin_89_8
GVMAG_S_3300005613_53
GVMAG_M_3300020165_35
Moniruzzaman_MM01_ERX552270_73
TARA_ARC_NCLDV_Bin_340_2
GVMAG_S_3300000115_46
Moniruzzaman_MM13_SRX803008_78
TARA_ARC_NCLDV_Bin_340_3
GVMAG_S_1041352_47
GVMAG_M_3300025727_45
GVMAG_M_3300017987_33
Moniruzzaman_MM06_SRX802073_13
YP 009052217 1 Viruses Varidnaviria Bamfordvirae Nucleocytoviricota Megaviricetes Algavirales Phycodnaviridae unclassified Phycodnaviridae Aureococcus anophagefferens virus
GVMAG_S_ERX555967_140
GVMAG_M_3300023210_4
GVMAG_S_ERX555935_167
Moniruzzaman_MM08_ERX552287_77
GVMAG_S_1073184_1
GVMAG_M_3300020194_30
GVMAG_M_3300009706_33
GVMAG_M_3300023179_117
TARA_ARC_NCLDV_Bin_172_2
Moniruzzaman_MM07_ERX552270_97
Moniruzzaman_MM07_SRX802073_62
GVMAG_S_1017745_26
GVMAG_S_1102114_101
GVMAG_M_3300023174_208
GVMAG_S_3300000576_28
GVMAG_S_1035082_32
GVMAG_S_ERX556032_80
GVMAG_M_3300021963_5
GVMAG_M_3300023179_31
GVMAG_M_3300013131_73
GVMAG_M_3300023174_6
GVMAG_S_3300013286_35
GVMAG_S_3300005662_175
GVMAG_S_1006664_89
GVMAG_M_3300009180_39
GVMAG_M_3300027747_41
GVMAG_M_3300010158_60
GVMAG_M_3300027969_16
GVMAG_S_1101165_163
GVMAG_M_3300006108_3
GVMAG_M_3300021137_6
GVMAG_M_3300023184_135
GVMAG_M_3300017967_12
GVMAG_S_3300000576_38
GVMAG_S_1016713_141
GVMAG_S_3300011013_78
GVMAG_M_3300020169_51
GVMAG_M_3300020187_69
GVMAG_S_ERX555997_44
GVMAG_M_3300020185_18
TARA_MED_NCLDV_Bin_341_9
GVMAG_M_3300023179_71
GVMAG_M_3300017989_16
GVMAG_M_3300017950_2
GVMAG_M_3300009159_48
GVMAG_M_3300009068_24
GVMAG_S_3300013006_147
GVMAG_M_3300023174_134
GVMAG_S_1101178_88
GVMAG_M_3300013093_49
GVMAG_M_3300027707_43
GVMAG_M_3300023184_120
GVMAG_M_3300020187_94
GVMAG_M_3300009441_23
GVMAG_S_ERX556045_64
GVMAG_M_3300024261_25
GVMAG_S_3300010368_67
GVMAG_M_3300025880_30
GVMAG_M_3300021375_17
GVMAG_M_3300025767_21
GVMAG_M_3300020185_80
GVMAG_M_3300003404_4
GVMAG_S_ERX555917_56
GVMAG_M_3300025874_67
GVMAG_M_3300021958_3
GVMAG_S_1039716_42
GVMAG_S_3300000929_49
GVMAG_M_3300009163_62
GVMAG_M_3300021079_11
GVMAG_M_3300023184_65
GVMAG_M_3300027892_54
GVMAG_M_3300024258_14
GVMAG_M_3300021961_61
GVMAG_M_3300020595_13
Moniruzzaman_MM01_ERX552302_47
GVMAG_S_ERX555967_69
GVMAG_S_3300012919_56
GVMAG_M_3300023174_107
GVMAG_M_3300023184_160
Viruses dsDNA viruses no RNA stage Phycodnaviridae environmental samples Organic Lake phycodnavirus 2 0
Viruses dsDNA viruses no RNA stage Phycodnaviridae environmental samples Organic Lake phycodnavirus 1 0
GVMAG_S_1102105_80
Moniruzzaman_MM01_SRX803008_24
GVMAG_S_ERX556022_25
YP 009173620 1 Viruses Varidnaviria Bamfordvirae Nucleocytoviricota Megaviricetes Algavirales Phycodnaviridae unclassified Phycodnaviridae Chrysochromulina ericina virus
GVMAG_M_3300009163_35
GVMAG_M_3300023174_116
GVMAG_S_ERX556060_80
YP 008052566 1 Viruses Varidnaviria Bamfordvirae Nucleocytoviricota Megaviricetes Algavirales Phycodnaviridae Prymnesiovirus unclassified Prymnesiovirus Phaeocystis globosa virus
Viruses dsDNA viruses no RNA stage Phycodnaviridae Prymnesiovirus unclassified Prymnesiovirus Phaeocystis globosa virus 0

B

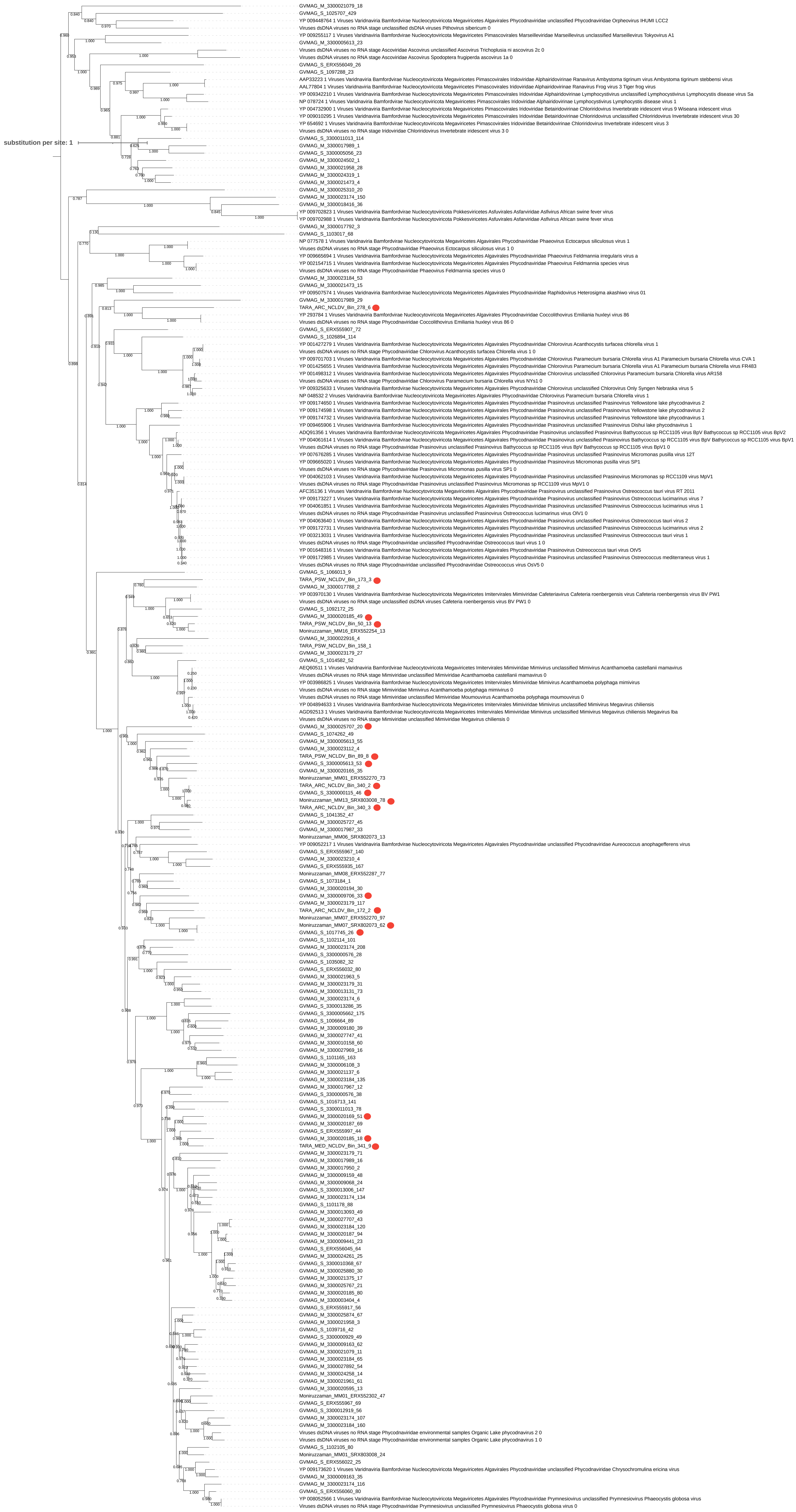
substitution per site: 1
GVMAG_M_3300021079_18
GVMAG_S_1025707_429
YP 009448764 1 Viruses Varidnaviria Bamfordvirae Nucleocytoviricota Megaviricetes Algavirales Phycodnaviridae unclassified Phycodnaviridae Orpheovirus IHUMI LCC2
Viruses dsDNA viruses no RNA stage unclassified dsDNA viruses Pithovirus sibericum 0
YP 009255117 1 Viruses Varidnaviria Bamfordvirae Nucleocytoviricota Megaviricetes Pimascovirales Marseilleviridae Marseillevirus unclassified Marseillevirus Tokyovirus A1
GVMAG_M_3300005613_23
Viruses dsDNA viruses no RNA stage Ascoviridae Ascovirus unclassified Ascovirus Trichoplusia ni ascovirus 2c 0
Viruses dsDNA viruses no RNA stage Ascoviridae Ascovirus Spodoptera frugiperda ascovirus 1a 0
GVMAG_S_ERX556049_26
GVMAG_S_1097288_23
AAP33223 1 Viruses Varidnaviria Bamfordvirae Nucleocytoviricota Megaviricetes Pimascovirales Iridoviridae Alphairidovirinae Ranavirus Ambystoma tigrinum virus Ambystoma tigrinum stebbensi virus
AAL77804 1 Viruses Varidnaviria Bamfordvirae Nucleocytoviricota Megaviricetes Pimascovirales Iridoviridae Alphairidovirinae Ranavirus Frog virus 3 Tiger frog virus
YP 009342210 1 Viruses Varidnaviria Bamfordvirae Nucleocytoviricota Megaviricetes Pimascovirales Iridoviridae Alphairidovirinae Lymphocystivirus unclassified Lymphocystivirus Lymphocystis disease virus Sa
NP 078724 1 Viruses Varidnaviria Bamfordvirae Nucleocytoviricota Megaviricetes Pimascovirales Iridoviridae Alphairidovirinae Lymphocystivirus Lymphocystis disease virus 1
YP 004732900 1 Viruses Varidnaviria Bamfordvirae Nucleocytoviricota Megaviricetes Pimascovirales Iridoviridae Betairidovirinae Chloriridovirus Invertebrate iridescent virus 9 Wiseana iridescent virus
YP 009010295 1 Viruses Varidnaviria Bamfordvirae Nucleocytoviricota Megaviricetes Pimascovirales Iridoviridae Betairidovirinae Chloriridovirus unclassified Chloriridovirus Invertebrate iridescent virus 30
YP 654692 1 Viruses Varidnaviria Bamfordvirae Nucleocytoviricota Megaviricetes Pimascovirales Iridoviridae Betairidovirinae Chloriridovirus Invertebrate iridescent virus 3
Viruses dsDNA viruses no RNA stage Iridoviridae Chloriridovirus Invertebrate iridescent virus 3 0
GVMAG_S_3300011013_114
GVMAG_M_3300017989_1
GVMAG_S_3300005056_23
GVMAG_M_3300024502_1
GVMAG_M_3300021958_28
GVMAG_M_3300024319_1
GVMAG_M_3300021473_4
GVMAG_M_3300025310_20
GVMAG_M_3300023174_150
GVMAG_M_3300018416_36
YP 009702823 1 Viruses Varidnaviria Bamfordvirae Nucleocytoviricota Pokkesviricetes Asfuvirales Asfarviridae Asfivirus African swine fever virus
YP 009702988 1 Viruses Varidnaviria Bamfordvirae Nucleocytoviricota Pokkesviricetes Asfuvirales Asfarviridae Asfivirus African swine fever virus
GVMAG_M_3300017792_3
GVMAG_S_1103017_68
NP 077578 1 Viruses Varidnaviria Bamfordvirae Nucleocytoviricota Megaviricetes Algavirales Phycodnaviridae Phaeovirus Ectocarpus siliculosus virus 1
Viruses dsDNA viruses no RNA stage Phycodnaviridae Phaeovirus Ectocarpus siliculosus virus 1 0
YP 009665694 1 Viruses Varidnaviria Bamfordvirae Nucleocytoviricota Megaviricetes Algavirales Phycodnaviridae Phaeovirus Feldmannia irregularis virus a
YP 002154715 1 Viruses Varidnaviria Bamfordvirae Nucleocytoviricota Megaviricetes Algavirales Phycodnaviridae Phaeovirus Feldmannia species virus
Viruses dsDNA viruses no RNA stage Phycodnaviridae Phaeovirus Feldmannia species virus 0
GVMAG_M_3300023184_53
GVMAG_M_3300021473_15
YP 009507574 1 Viruses Varidnaviria Bamfordvirae Nucleocytoviricota Megaviricetes Algavirales Phycodnaviridae Raphidovirus Heterosigma akashiwo virus 01
GVMAG_M_3300017989_29
TARA_ARC_NCLDV_Bin_278_6
YP 293784 1 Viruses Varidnaviria Bamfordvirae Nucleocytoviricota Megaviricetes Algavirales Phycodnaviridae Coccolithovirus Emiliania huxleyi virus 86
Viruses dsDNA viruses no RNA stage Phycodnaviridae Coccolithovirus Emiliania huxleyi virus 86 0
GVMAG_S_ERX555907_72
GVMAG_S_1026894_114
YP 001427279 1 Viruses Varidnaviria Bamfordvirae Nucleocytoviricota Megaviricetes Algavirales Phycodnaviridae Chlorovirus Acanthocystis turfacea chlorella virus 1
Viruses dsDNA viruses no RNA stage Phycodnaviridae Chlorovirus Acanthocystis turfacea Chlorella virus 1 0
YP 009701703 1 Viruses Varidnaviria Bamfordvirae Nucleocytoviricota Megaviricetes Algavirales Phycodnaviridae Chlorovirus Paramecium bursaria Chlorella virus A1 Paramecium bursaria Chlorella virus CVA 1
YP 001425655 1 Viruses Varidnaviria Bamfordvirae Nucleocytoviricota Megaviricetes Algavirales Phycodnaviridae Chlorovirus Paramecium bursaria Chlorella virus A1 Paramecium bursaria Chlorella virus FR483
YP 001498312 1 Viruses Varidnaviria Bamfordvirae Nucleocytoviricota Megaviricetes Algavirales Phycodnaviridae Chlorovirus unclassified Chlorovirus Paramecium bursaria Chlorella virus AR158
Viruses dsDNA viruses no RNA stage Phycodnaviridae Chlorovirus Paramecium bursaria Chlorella virus NYs1 0
YP 009325633 1 Viruses Varidnaviria Bamfordvirae Nucleocytoviricota Megaviricetes Algavirales Phycodnaviridae Chlorovirus unclassified Chlorovirus Only Syngen Nebraska virus 5
NP 048532 2 Viruses Varidnaviria Bamfordvirae Nucleocytoviricota Megaviricetes Algavirales Phycodnaviridae Chlorovirus Paramecium bursaria Chlorella virus 1
YP 009174650 1 Viruses Varidnaviria Bamfordvirae Nucleocytoviricota Megaviricetes Algavirales Phycodnaviridae Prasinovirus unclassified Prasinovirus Yellowstone lake phycodnavirus 2
YP 009174598 1 Viruses Varidnaviria Bamfordvirae Nucleocytoviricota Megaviricetes Algavirales Phycodnaviridae Prasinovirus unclassified Prasinovirus Yellowstone lake phycodnavirus 2
YP 009174732 1 Viruses Varidnaviria Bamfordvirae Nucleocytoviricota Megaviricetes Algavirales Phycodnaviridae Prasinovirus unclassified Prasinovirus Yellowstone lake phycodnavirus 1
YP 009465906 1 Viruses Varidnaviria Bamfordvirae Nucleocytoviricota Megaviricetes Algavirales Phycodnaviridae Prasinovirus unclassified Prasinovirus Dishui lake phycodnavirus 1
ADQ91356 1 Viruses Varidnaviria Bamfordvirae Nucleocytoviricota Megaviricetes Algavirales Phycodnaviridae Prasinovirus unclassified Prasinovirus Bathycoccus sp RCC1105 virus BpV Bathycoccus sp RCC1105 virus BpV2
YP 004061614 1 Viruses Varidnaviria Bamfordvirae Nucleocytoviricota Megaviricetes Algavirales Phycodnaviridae Prasinovirus unclassified Prasinovirus Bathycoccus sp RCC1105 virus BpV Bathycoccus sp RCC1105 virus BpV1
Viruses dsDNA viruses no RNA stage Phycodnaviridae Prasinovirus unclassified Prasinovirus Bathycoccus sp RCC1105 virus BpV Bathycoccus sp RCC1105 virus BpV1 0
YP 007676285 1 Viruses Varidnaviria Bamfordvirae Nucleocytoviricota Megaviricetes Algavirales Phycodnaviridae Prasinovirus unclassified Prasinovirus Micromonas pusilla virus 12T
YP 009665020 1 Viruses Varidnaviria Bamfordvirae Nucleocytoviricota Megaviricetes Algavirales Phycodnaviridae Prasinovirus Micromonas pusilla virus SP1
Viruses dsDNA viruses no RNA stage Phycodnaviridae Prasinovirus Micromonas pusilla virus SP1 0
YP 004062103 1 Viruses Varidnaviria Bamfordvirae Nucleocytoviricota Megaviricetes Algavirales Phycodnaviridae Prasinovirus unclassified Prasinovirus Micromonas sp RCC1109 virus MpV1
Viruses dsDNA viruses no RNA stage Phycodnaviridae Prasinovirus unclassified Prasinovirus Micromonas sp RCC1109 virus MpV1 0
AFC35136 1 Viruses Varidnaviria Bamfordvirae Nucleocytoviricota Megaviricetes Algavirales Phycodnaviridae Prasinovirus unclassified Prasinovirus Ostreococcus tauri virus RT 2011
YP 009173227 1 Viruses Varidnaviria Bamfordvirae Nucleocytoviricota Megaviricetes Algavirales Phycodnaviridae Prasinovirus unclassified Prasinovirus Ostreococcus lucimarinus virus 7
YP 004061851 1 Viruses Varidnaviria Bamfordvirae Nucleocytoviricota Megaviricetes Algavirales Phycodnaviridae Prasinovirus unclassified Prasinovirus Ostreococcus lucimarinus virus 1
Viruses dsDNA viruses no RNA stage Phycodnaviridae Prasinovirus unclassified Prasinovirus Ostreococcus lucimarinus virus OlV1 0
YP 004063640 1 Viruses Varidnaviria Bamfordvirae Nucleocytoviricota Megaviricetes Algavirales Phycodnaviridae Prasinovirus unclassified Prasinovirus Ostreococcus tauri virus 2
YP 009172731 1 Viruses Varidnaviria Bamfordvirae Nucleocytoviricota Megaviricetes Algavirales Phycodnaviridae Prasinovirus unclassified Prasinovirus Ostreococcus lucimarinus virus 2
YP 003213031 1 Viruses Varidnaviria Bamfordvirae Nucleocytoviricota Megaviricetes Algavirales Phycodnaviridae Prasinovirus unclassified Prasinovirus Ostreococcus tauri virus 1
Viruses dsDNA viruses no RNA stage Phycodnaviridae Prasinovirus unclassified Phycodnaviridae Ostreococcus tauri virus 1 0
YP 001648316 1 Viruses Varidnaviria Bamfordvirae Nucleocytoviricota Megaviricetes Algavirales Phycodnaviridae Prasinovirus Ostreococcus tauri virus OtV5
YP 009172985 1 Viruses Varidnaviria Bamfordvirae Nucleocytoviricota Megaviricetes Algavirales Phycodnaviridae Prasinovirus unclassified Prasinovirus Ostreococcus mediterraneus virus 1
Viruses dsDNA viruses no RNA stage Phycodnaviridae unclassified Phycodnaviridae Ostreococcus virus OsV5 0
GVMAG_S_1066013_9
TARA_PSW_NCLDV_Bin_173_3
GVMAG_M_3300017788_2
YP 003970130 1 Viruses Varidnaviria Bamfordvirae Nucleocytoviricota Megaviricetes Imitervirales Mimiviridae Cafeteriavirus Cafeteria roenbergensis virus BV PW1
Viruses dsDNA viruses no RNA stage unclassified dsDNA viruses Cafeteria roenbergensis virus BV PW1 0
GVMAG_S_1092172_25
GVMAG_M_3300020185_49
TARA_PSW_NCLDV_Bin_50_13
Moniruzzaman_MM16_ERX552254_13
GVMAG_M_3300022916_4
TARA_PSW_NCLDV_Bin_158_1
GVMAG_M_3300023179_27
GVMAG_S_1014582_52
AEQ60511 1 Viruses Varidnaviria Bamfordvirae Nucleocytoviricota Megaviricetes Imitervirales Mimiviridae Mimivirus unclassified Mimivirus Acanthamoeba castellanii mamavirus
Viruses dsDNA viruses no RNA stage Mimiviridae unclassified Mimiviridae Acanthamoeba castellanii mamavirus 0
YP 003986825 1 Viruses Varidnaviria Bamfordvirae Nucleocytoviricota Megaviricetes Imitervirales Mimiviridae Mimivirus Acanthamoeba polyphaga mimivirus
Viruses dsDNA viruses no RNA stage Mimiviridae Mimivirus Acanthamoeba polyphaga mimivirus 0
Viruses dsDNA viruses no RNA stage Mimiviridae unclassified Mimiviridae Moumouvirus Acanthamoeba polyphaga moumouvirus 0
YP 004894633 1 Viruses Varidnaviria Bamfordvirae Nucleocytoviricota Megaviricetes Imitervirales Mimiviridae Mimivirus unclassified Mimivirus Megavirus chiliensis
AGD92513 1 Viruses Varidnaviria Bamfordvirae Nucleocytoviricota Megaviricetes Imitervirales Mimiviridae Mimivirus unclassified Mimivirus Megavirus chiliensis Megavirus lba
Viruses dsDNA viruses no RNA stage Mimiviridae unclassified Mimiviridae Megavirus chiliensis 0
GVMAG_M_3300025707_20
GVMAG_S_1074262_49
GVMAG_M_3300005613_55
GVMAG_M_3300023112_4
TARA_PSW_NCLDV_Bin_89_8
GVMAG_S_3300005613_53
GVMAG_M_3300020165_35
Moniruzzaman_MM01_ERX552270_73
TARA_ARC_NCLDV_Bin_340_2
GVMAG_S_3300000115_46
Moniruzzaman_MM13_SRX803008_78
TARA_ARC_NCLDV_Bin_340_3
GVMAG_S_1041352_47
GVMAG_M_3300025727_45
GVMAG_M_3300017987_33
Moniruzzaman_MM06_SRX802073_13
YP 009052217 1 Viruses Varidnaviria Bamfordvirae Nucleocytoviricota Megaviricetes Algavirales Phycodnaviridae unclassified Phycodnaviridae Aureococcus anophagefferens virus
GVMAG_S_ERX555967_140
GVMAG_M_3300023210_4
GVMAG_S_ERX555935_167
Moniruzzaman_MM08_ERX552287_77
GVMAG_S_1073184_1
GVMAG_M_3300020194_30
GVMAG_M_3300009706_33
GVMAG_M_3300023179_117
TARA_ARC_NCLDV_Bin_172_2
Moniruzzaman_MM07_ERX552270_97
Moniruzzaman_MM07_SRX802073_62
GVMAG_S_1017745_26
GVMAG_S_1102114_101
GVMAG_M_3300023174_208
GVMAG_S_3300000576_28
GVMAG_S_1035082_32
GVMAG_S_ERX556032_80
GVMAG_M_3300021963_5
GVMAG_M_3300023179_31
GVMAG_M_3300013131_73
GVMAG_M_3300023174_6
GVMAG_S_3300013286_35
GVMAG_S_3300005662_175
GVMAG_S_1006664_89
GVMAG_M_3300009180_39
GVMAG_M_3300027747_41
GVMAG_M_3300010158_60
GVMAG_M_3300027969_16
GVMAG_S_1101165_163
GVMAG_M_3300006108_3
GVMAG_M_3300021137_6
GVMAG_M_3300023184_135
GVMAG_M_3300017967_12
GVMAG_S_3300000576_38
GVMAG_S_1016713_141
GVMAG_S_3300011013_78
GVMAG_M_3300020169_51
GVMAG_M_3300020187_69
GVMAG_S_ERX555997_44
GVMAG_M_3300020185_18
TARA_MED_NCLDV_Bin_341_9
GVMAG_M_3300023179_71
GVMAG_M_3300017989_16
GVMAG_M_3300017950_2
GVMAG_M_3300009159_48
GVMAG_M_3300009068_24
GVMAG_S_3300013006_147
GVMAG_M_3300023174_134
GVMAG_S_1101178_88
GVMAG_M_3300013093_49
GVMAG_M_3300027707_43
GVMAG_M_3300023184_120
GVMAG_M_3300020187_94
GVMAG_M_3300009441_23
GVMAG_S_ERX556045_64
GVMAG_M_3300024261_25
GVMAG_S_3300010368_67
GVMAG_M_3300025880_30
GVMAG_M_3300021375_17
GVMAG_M_3300025767_21
GVMAG_M_3300020185_80
GVMAG_M_3300003404_4
GVMAG_S_ERX555917_56
GVMAG_M_3300025874_67
GVMAG_M_3300021958_3
GVMAG_S_1039716_42
GVMAG_S_3300000929_49
GVMAG_M_3300009163_62
GVMAG_M_3300021079_11
GVMAG_M_3300023184_65
GVMAG_M_3300027892_54
GVMAG_M_3300024258_14
GVMAG_M_3300021961_61
GVMAG_M_3300020595_13
Moniruzzaman_MM01_ERX552302_47
GVMAG_S_ERX555967_69
GVMAG_S_3300012919_56
GVMAG_M_3300023174_107
GVMAG_M_3300023184_160
Viruses dsDNA viruses no RNA stage Phycodnaviridae environmental samples Organic Lake phycodnavirus 2 0
Viruses dsDNA viruses no RNA stage Phycodnaviridae environmental samples Organic Lake phycodnavirus 1 0
GVMAG_S_1102105_80
Moniruzzaman_MM01_SRX803008_24
GVMAG_S_ERX556022_25
YP 009173620 1 Viruses Varidnaviria Bamfordvirae Nucleocytoviricota Megaviricetes Algavirales Phycodnaviridae unclassified Phycodnaviridae Chrysochromulina ericina virus
GVMAG_M_3300009163_35
GVMAG_M_3300023174_116
GVMAG_S_ERX556060_80
YP 008052566 1 Viruses Varidnaviria Bamfordvirae Nucleocytoviricota Megaviricetes Algavirales Phycodnaviridae Prymnesiovirus unclassified Prymnesiovirus Phaeocystis globosa virus
Viruses dsDNA viruses no RNA stage Phycodnaviridae Prymnesiovirus unclassified Prymnesiovirus Phaeocystis globosa virus 0
