# Supplementary material for: Discovery of Viral Myosin Genes With Complex Evolutionary History Within Plankton
Source: Front Microbiol. 2021 Jun 7;12:683294. doi: 10.3389/fmicb.2021.683294 (PMC8215601; doi:10.3389/fmicb.2021.683294)
Supplement: Supplementary file 7 [file Data_Sheet_3.PDF]

Supplementary Figure 3 | Phylgenetic trees of myosin sequences. **(A)** Phylogeny of myosin from NCLDVs and eukaryotic lineages (gappy/IQ-TREE). The tree was built from the multiple sequence alignment (658 sites) of 286 myosin head domain sequences. Numerical values represent the non-parametric bootstrap values for the branch support. The labels of virmyosins are marked with red circle. We consider myosin-II as an outgroup to root this tree. The LG+R10 substitution model was selected for the best model for tree reconstruction. **(B)** Phylogeny of myosin from NCLDVs and eukaryotic lineages (strict/IQ-TREE). The tree was built from the multiple sequence alignment (411 sites) of 286 myosin head domain sequences. Numerical values represent the non-parametric bootstrap values for the branch support. The labels of virmyosins are marked with red circle. We consider myosin-II as an outgroup to root this tree. The LG+F+R10 substitution model was selected for the best model for tree reconstruction. **(C)** Phylogeny of myosin from NCLDVs and eukaryotic lineages (gappy/RAxML). The tree was built from the multiple sequence alignment (658 sites) of 286 myosin head domain sequences. Numerical values represent the non-parametric bootstrap values for the branch support. The labels of virmyosins are marked with red circle. We consider myosin-II as an outgroup to root this tree. The PROTGAMMAIAUTO substitution model was selected for the best model for tree reconstruction. **(D)** Phylogenetic tree of myosins (strict/RAxML). The tree was built from the multiple sequence alignment (411 sites) of 286 myosins. Numerical values represent the non-parametric bootstrap values for the branch support. The labels of virmyosins are marked with red circle. We consider myosin-II as outgroup to root this tree. The PROTGAMMAIAUTO substitution model was selected for the best model for tree reconstruction. **(E)** Phylogenetic tree of myosins (gappy/IQ-TREE). The tree was built from the multiple sequence alignment (658 sites) of 286 myosins. Numerical values represent the transfer bootstrap expectation for the branch support. The labels of virmyosins are marked with red circle. We consider myosinII as outgroup to root this tree. **(F)** Phylogeny of myosin from NCLDVs and close relatives in eukaryotic lineages. The tree was built from the multiple sequence alignment (688 sites) of 81 myosin head domain sequences. Numerical values represent the non-parametric bootstrap values for the branch support. The labels of virmyosins are marked with red circle. We consider myosin-II of *Nasonia vitripennis* and *Neurospora crassa* as an outgroup to root this tree. The LG+F+R7substitution model was selected for the best model for tree reconstruction. **(G)** Phylogeny of myosin from NCLDVs and close relatives in eukaryotic lineages. The tree was built from the multiple sequence alignment (688 sites) of 81 myosins. Numerical values represent the transfer bootstrap expectation for the branch support. The labels of virmyosins are marked with red circle. We consider

myosin-II of *Nasonia vitripennis* and *Neurospora crassa* as outgroup to root this tree. **(H)** Phylogeny of myosin from NCLDVs and close relatives in eukaryotic lineages. The tree was built from the multiple sequence alignment (653 sites) of 208 myosin head domain sequences. Numerical values represent the non-parametric bootstrap values for the branch support. The labels of virmyosins are marked with red circle. We consider myosin-II of Nasonia vitripennis and Neurospora crassa as an outgroup to root this tree. The LG+F+R10 substitution model was selected for the best model for tree reconstruction. **(I)** Phylogeny of myosin from NCLDVs and close relatives in eukaryotic lineages. The tree was built from the multiple sequence alignment (653 sites) of 208 myosins. Numerical values represent the transfer bootstrap expectation for the branch support. The labels of virmyosins are marked with red circle. We consider myosin-II of Nasonia vitripennis and Neurospora crassa as outgroup to root this tree

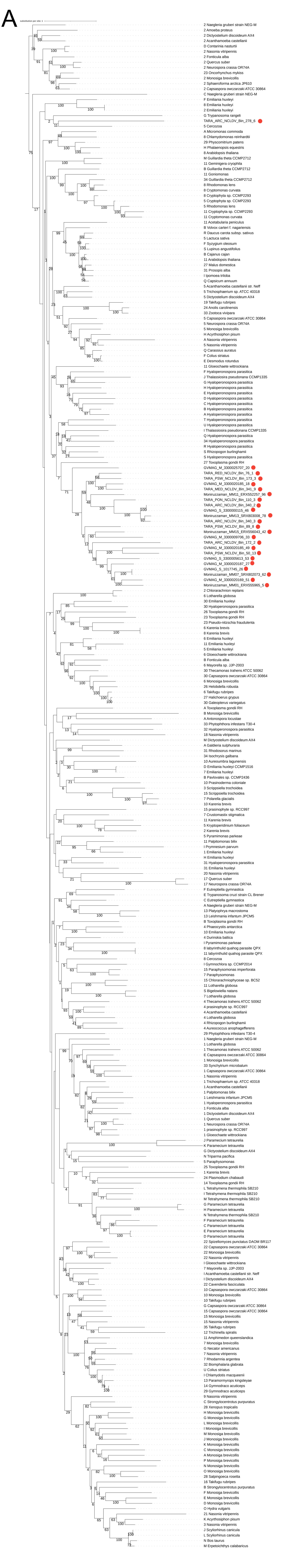
A
substitution per site: 1
2 Naegleria gruberi strain NEG-M
2 Amoeba proteus
2 Dictyostelium discoideum AX4
2 Acanthamoeba castellanii
D Contarinia nasturtii
2 Nasonia vitripennis
2 Fonticula alba
2 Quercus suber
2 Neurospora crassa OR74A
23 Oncorhynchus mykiss
2 Monosiga brevicollis
2 Sphaeroforma arctica JP610
2 Capsaspora owczarzaki ATCC 30864
C Naegleria gruberi strain NEG-M
F Emiliania huxleyi
B Emiliania huxleyi
2 Emiliania huxleyi
G Trypanosoma rangeli
TARA_ARC_NCLDV_Bin_278_6
5 Cercozoa
A Micromonas commoda
8 Chlamydomonas reinhardtii
29 Physcomitrium patens
H Phalaenopsis equestris
8 Arabidopsis thaliana
M Guillardia theta CCMP2712
11 Geminigera cryophila
B Guillardia theta CCMP2712
11 Goniomonas
34 Guillardia theta CCMP2712
8 Rhodomonas lens
8 Cryptomonas curvata
8 Cryptophyta sp. CCMP2293
5 Cryptophyta sp. CCMP2293
5 Rhodomonas lens
11 Cryptophyta sp. CCMP2293
11 Cryptomonas curvata
11 Acetabularia peniculus
B Volvox carteri f. nagariensis
R Daucus carota subsp. sativus
5 Lactuca sativa
F Syzygium oleosum
S Lupinus angustifolius
8 Cajanus cajan
11 Arabidopsis thaliana
27 Malus domestica
31 Prosopis alba
I Ipomoea triloba
Q Capsicum annuum
5 Acanthamoeba castellanii str. Neff
5 Trichosphaerium sp. ATCC 40318
5 Dictyostelium discoideum AX4
19 Takifugu rubripes
24 Anolis carolinensis
33 Zootoca vivipara
5 Capsaspora owczarzaki ATCC 30864
5 Neurospora crassa OR74A
5 Monosiga brevicollis
H Acyrthosiphon pisum
A Nasonia vitripennis
2 Nasonia vitripennis
Q Carassius auratus
F Colius striatus
E Desmodus rotundus
11 Gloeochaete wittrockiana
F Hyaloperonospora parasitica
J Thalassiosira pseudonana CCMP1335
G Hyaloperonospora parasitica
H Hyaloperonospora parasitica
E Hyaloperonospora parasitica
D Hyaloperonospora parasitica
C Hyaloperonospora parasitica
B Hyaloperonospora parasitica
A Hyaloperonospora parasitica
T Hyaloperonospora parasitica
U Hyaloperonospora parasitica
I Thalassiosira pseudonana CCMP1335
Q Hyaloperonospora parasitica
34 Hyaloperonospora parasitica
R Hyaloperonospora parasitica
S Rhizopogon burlinghamii
S Hyaloperonospora parasitica
27 Toxoplasma gondii RH
GVMAG_M_3300025707_20
TARA_RED_NCLDV_Bin_76_1
TARA_PSW_NCLDV_Bin_173_3
GVMAG_M_3300020185_18
TARA_MED_NCLDV_Bin_341_9
Moniruzzaman_MM11_ERX552257_96
TARA_PON_NCLDV_Bin_110_3
TARA_ARC_NCLDV_Bin_340_2
GVMAG_S_3300000115_46
Moniruzzaman_MM13_SRX803008_78
TARA_ARC_NCLDV_Bin_340_3
TARA_PSW_NCLDV_Bin_89_8
Moniruzzaman_MM15_ERX556043_42
GVMAG_M_3300009706_33
TARA_ARC_NCLDV_Bin_172_2
GVMAG_M_3300020185_49
TARA_PSW_NCLDV_Bin_50_13
GVMAG_S_3300005613_53
GVMAG_M_3300020187_27
GVMAG_S_1017745_26
Moniruzzaman_MM07_SRX802073_62
GVMAG_M_3300020189_51
Moniruzzaman_MM01_ERX555965_5
2 Chlorarachnion reptans
6 Lotharella globosa
30 Emiliania huxleyi
30 Hyaloperonospora parasitica
26 Toxoplasma gondii RH
23 Toxoplasma gondii RH
23 Pseudo-nitzschia fraudulenta
6 Karenia brevis
8 Karenia brevis
6 Emiliania huxleyi
11 Emiliania huxleyi
5 Emiliania huxleyi
6 Gloeochaete wittrockiana
B Fonticula alba
6 Mayorella sp. JJP-2003
30 Thecamonas trahens ATCC 50062
30 Capsaspora owczarzaki ATCC 30864
6 Monosiga brevicollis
26 Helobdella robusta
6 Takifugu rubripes
27 Halichoerus grypus
30 Galeopterus variegatus
A Toxoplasma gondii RH
B Monosiga brevicollis
A Antonospora locustae
33 Phytophthora infestans T30-4
32 Hyaloperonospora parasitica
18 Nasonia vitripennis
M Dictyostelium discoideum AX4
A Galdieria sulphuraria
31 Rhodosorus marinus
34 Isochrysis galbana
10 Aureoumbra lagunensis
D Emiliania huxleyi CCMP1516
7 Emiliania huxleyi
B Pavlovales sp. CCMP2436
10 Prasinoderma coloniale
3 Scrippsiella trochoidea
15 Scrippsiella trochoidea
7 Polarella glacialis
10 Karenia brevis
5 prasinophyte sp. RCC997
7 Crustomastix stigmatica
11 Karenia brevis
5 Kryptoperidinium foliaceum
2 Karenia brevis
5 Pyramimonas parkeae
11 Palpitomonas bilix
I Prymnesium parvum
I Emiliania huxleyi
H Emiliania huxleyi
31 Hyaloperonospora parasitica
31 Emiliania huxleyi
20 Nasonia vitripennis
17 Quercus suber
17 Neurospora crassa OR74A
F Eutreptiella gymnastica
E Trypanosoma cruzi strain CL Brener
C Eutreptiella gymnastica
A Naegleria gruberi strain NEG-M
13 Platyophrya macrostoma
13 Leishmania infantum JPCM5
B Toxoplasma gondii RH
4 Phaeocystis antarctica
10 Emiliania huxleyi
4 Durinskia baltica
I Pyramimonas parkeae
8 labyrinthulid quahog parasite QPX
11 labyrinthulid quahog parasite QPX
8 Cercozoa
I Gymnochlora sp. CCMP2014
15 Paraphysomonas imperforata
7 Paraphysomonas
15 Chlorarachniophyceae sp. BC52
11 Lotharella globosa
S Bigelowiella natans
7 Lotharella globosa
4 Thecamonas trahens ATCC 50062
4 prasinophyte sp. RCC997
4 Acanthamoeba castellanii
4 Lotharella globosa
4 Rhizopogon burlinghamii
4 Aureococcus anophagefferens
29 Phytophthora infestans T30-4
1 Naegleria gruberi strain NEG-M
1 Lotharella globosa
1 Thecamonas trahens ATCC 50062
E Capsaspora owczarzaki ATCC 30864
1 Monosiga brevicollis
33 Synchytrium microbalum
1 Capsaspora owczarzaki ATCC 30864
1 Nasonia vitripennis
1 Trichosphaerium sp. ATCC 40318
1 Acanthamoeba castellanii
1 Palpitomonas bilix
1 Leishmania infantum JPCM5
1 Hyaloperonospora parasitica
1 Fonticula alba
1 Dictyostelium discoideum AX4
1 Quercus suber
1 Neurospora crassa OR74A
1 prasinophyte sp. RCC997
1 Gloeochaete wittrockiana
J Paramecium tetraurelia
K Paramecium tetraurelia
G Dictyostelium discoideum AX4
N Triparma pacifica
5 Paraphysomonas
25 Toxoplasma gondii RH
1 Karenia brevis
24 Plasmodium chabaudi
14 Toxoplasma gondii RH
L Tetrahymena thermophila SB210
I Tetrahymena thermophila SB210
M Tetrahymena thermophila SB210
G Paramecium tetraurelia
H Paramecium tetraurelia
N Tetrahymena thermophila SB210
F Paramecium tetraurelia
C Paramecium tetraurelia
E Paramecium tetraurelia
D Paramecium tetraurelia
22 Spizellomyces punctatus DAOM BR117
22 Capsaspora owczarzaki ATCC 30864
22 Monosiga brevicollis
22 Nasonia vitripennis
I Gloeochaete wittrockiana
7 Mayorella sp. JJP-2003
I Acanthamoeba castellanii str. Neff
I Dictyostelium discoideum AX4
22 Cavenderia fasciculata
10 Capsaspora owczarzaki ATCC 30864
10 Monosiga brevicollis
10 Takifugu rubripes
G Capsaspora owczarzaki ATCC 30864
15 Capsaspora owczarzaki ATCC 30864
15 Monosiga brevicollis
15 Nasonia vitripennis
35 Takifugu rubripes
12 Trichinella spiralis
11 Amphimedon queenslandica
7 Monosiga brevicollis
G Necator americanus
7 Nasonia vitripennis
7 Rhodamnia argentea
32 Biomphalaria glabrata
U Colius striatus
I Chlamydotis macqueenii
13 Paramormyrops kingsleyae
14 Gymnodraco acuticeps
29 Gymnodraco acuticeps
9 Nasonia vitripennis
C Strongylocentrotus purpuratus
28 Xenopus tropicalis
H Monosiga brevicollis
G Monosiga brevicollis
L Monosiga brevicollis
I Monosiga brevicollis
M Monosiga brevicollis
J Monosiga brevicollis
K Monosiga brevicollis
C Monosiga brevicollis
A Monosiga brevicollis
P Monosiga brevicollis
N Monosiga brevicollis
O Monosiga brevicollis
28 Salpingoeca rosetta
16 Takifugu rubripes
B Strongylocentrotus purpuratus
F Monosiga brevicollis
E Monosiga brevicollis
D Monosiga brevicollis
O Hydra vulgaris
21 Nasonia vitripennis
K Acyrthosiphon pisum
3 Nasonia vitripennis
J Scyliorhinus canicula
L Scyliorhinus canicula
N Bos taurus
M Erpetoichthys calabaricus

B

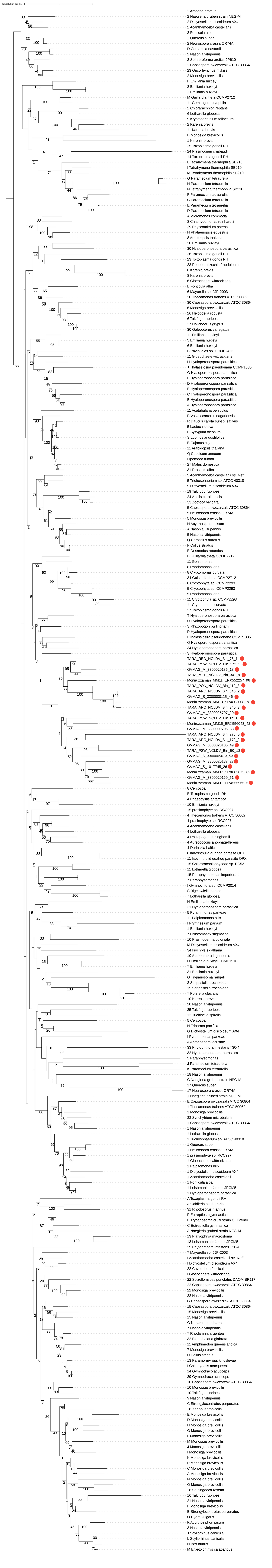

C

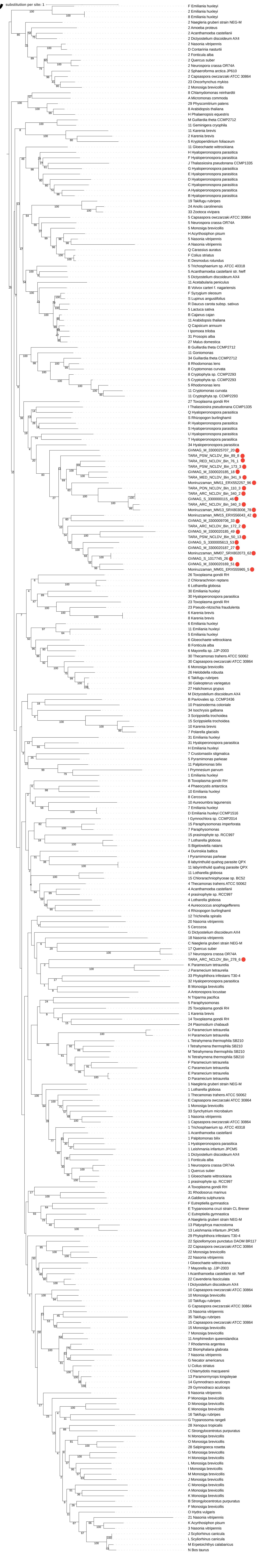

D

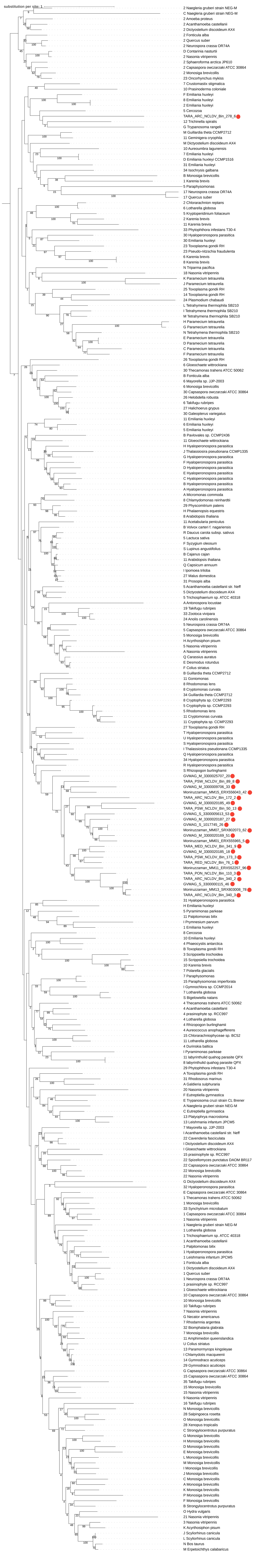
substitution per site: 1
2 Naegleria gruberi strain NEG-M
C Naegleria gruberi strain NEG-M
2 Amoeba proteus
2 Acanthamoeba castellanii
2 Dictyostelium discoideum AX4
2 Fonticula alba
2 Quercus suber
2 Neurospora crassa OR74A
D Contarinia nasturtii
2 Nasonia vitripennis
2 Sphaeroforma arctica JP610
2 Capsaspora owczarzaki ATCC 30864
2 Monosiga brevicollis
23 Oncorhynchus mykiss
7 Crustomastix stigmatica
10 Prasinoderma coloniale
F Emiliania huxleyi
8 Emiliania huxleyi
2 Emiliania huxleyi
5 Cercozoa
TARA_ARC_NCLDV_Bin_278_6
12 Trichinella spiralis
G Trypanosoma rangeli
M Guillardia theta CCMP2712
11 Geminigera cryophila
M Dictyostelium discoideum AX4
10 Aureoumbra lagunensis
7 Emiliania huxleyi
D Emiliania huxleyi CCMP1516
31 Emiliania huxleyi
34 Isochrysis galbana
B Monosiga brevicollis
1 Karenia brevis
5 Paraphysomonas
17 Neurospora crassa OR74A
17 Quercus suber
2 Chlorarachnion reptans
6 Lotharella globosa
5 Kryptoperidinium foliaceum
2 Karenia brevis
11 Karenia brevis
33 Phytophthora infestans T30-4
30 Hyaloperonospora parasitica
30 Emiliania huxleyi
23 Toxoplasma gondii RH
23 Pseudo-nitzschia fraudulenta
6 Karenia brevis
8 Karenia brevis
N Triparma pacifica
18 Nasonia vitripennis
K Paramecium tetraurelia
J Paramecium tetraurelia
25 Toxoplasma gondii RH
14 Toxoplasma gondii RH
24 Plasmodium chabaudi
L Tetrahymena thermophila SB210
I Tetrahymena thermophila SB210
M Tetrahymena thermophila SB210
H Paramecium tetraurelia
G Paramecium tetraurelia
N Tetrahymena thermophila SB210
E Paramecium tetraurelia
D Paramecium tetraurelia
C Paramecium tetraurelia
F Paramecium tetraurelia
26 Toxoplasma gondii RH
6 Gloeochaete wittrockiana
30 Thecamonas trahens ATCC 50062
B Fonticula alba
6 Mayorella sp. JJP-2003
6 Monosiga brevicollis
30 Capsaspora owczarzaki ATCC 30864
26 Helobdella robusta
6 Takifugu rubripes
27 Halichoerus grypus
30 Galeopterus variegatus
11 Emiliania huxleyi
6 Emiliania huxleyi
5 Emiliania huxleyi
B Pavlovales sp. CCMP2436
11 Gloeochaete wittrockiana
H Hyaloperonospora parasitica
J Thalassiosira pseudonana CCMP1335
G Hyaloperonospora parasitica
F Hyaloperonospora parasitica
D Hyaloperonospora parasitica
E Hyaloperonospora parasitica
C Hyaloperonospora parasitica
B Hyaloperonospora parasitica
A Hyaloperonospora parasitica
A Micromonas commoda
8 Chlamydomonas reinhardtii
29 Physcomitrium patens
H Phalaenopsis equestris
8 Arabidopsis thaliana
11 Acetabularia peniculus
B Volvox carteri f. nagariensis
R Daucus carota subsp. sativus
S Lactuca sativa
F Syzygium oleosum
S Lupinus angustifolius
B Cajanus cajan
11 Arabidopsis thaliana
Q Capsicum annuum
I Ipomoea triloba
27 Malus domestica
31 Prosopis alba
5 Acanthamoeba castellanii str. Neff
5 Dictyostelium discoideum AX4
5 Trichosphaerium sp. ATCC 40318
A Antonospora locustae
19 Takifugu rubripes
33 Zootoca vivipara
24 Anolis carolinensis
5 Neurospora crassa OR74A
5 Capsaspora owczarzaki ATCC 30864
5 Monosiga brevicollis
H Acyrthosiphon pisum
5 Nasonia vitripennis
A Nasonia vitripennis
Q Carassius auratus
E Desmodus rotundus
F Colius striatus
B Guillardia theta CCMP2712
11 Goniomonas
8 Rhodomonas lens
8 Cryptomonas curvata
34 Guillardia theta CCMP2712
8 Cryptophyta sp. CCMP2293
5 Cryptophyta sp. CCMP2293
5 Rhodomonas lens
11 Cryptomonas curvata
11 Cryptophyta sp. CCMP2293
27 Toxoplasma gondii RH
T Hyaloperonospora parasitica
U Hyaloperonospora parasitica
S Hyaloperonospora parasitica
I Thalassiosira pseudonana CCMP1335
Q Hyaloperonospora parasitica
34 Hyaloperonospora parasitica
R Hyaloperonospora parasitica
S Rhizopogon burlinghamii
GVMAG_M_3300025707_20
TARA_PSW_NCLDV_Bin_89_8
GVMAG_M_3300009706_33
Moniruzzaman_MM15_ERX556043_42
TARA_ARC_NCLDV_Bin_172_2
GVMAG_M_3300020185_49
TARA_PSW_NCLDV_Bin_50_13
GVMAG_S_3300005613_53
GVMAG_M_3300020187_27
GVMAG_S_1017745_26
Moniruzzaman_MM07_SRX802073_62
GVMAG_M_3300020169_51
Moniruzzaman_MM01_ERX555965_5
TARA_MED_NCLDV_Bin_341_9
GVMAG_M_3300020185_18
TARA_PSW_NCLDV_Bin_173_3
TARA_RED_NCLDV_Bin_76_1
Moniruzzaman_MM11_ERX552257_96
TARA_PON_NCLDV_Bin_110_3
TARA_ARC_NCLDV_Bin_340_2
GVMAG_S_3300000115_46
Moniruzzaman_MM13_SRX803006_78
TARA_ARC_NCLDV_Bin_340_3
31 Hyaloperonospora parasitica
H Emiliania huxleyi
5 Pyramimonas parkeae
11 Palpitomonas bilix
I Prymnesium parvum
1 Emiliania huxleyi
8 Cercozoa
10 Emiliania huxleyi
4 Phaeocystis antarctica
B Toxoplasma gondii RH
3 Scrippsiella trochoidea
15 Scrippsiella trochoidea
10 Karenia brevis
7 Polarella glacialis
7 Paraphysomonas
15 Paraphysomonas imperforata
I Gymnochlora sp. CCMP2014
7 Lotharella globosa
5 Bigelowiella natans
4 Thecamonas trahens ATCC 50062
4 Acanthamoeba castellanii
4 prasinophyte sp. RCC997
4 Lotharella globosa
4 Rhizopogon burlinghamii
4 Aureococcus anophagefferens
15 Chloarachniophyceae sp. BC52
11 Lotharella globosa
4 Durinskia baltica
I Pyramimonas parkeae
11 labyrinthulid quahog parasite QPX
8 labyrinthulid quahog parasite QPX
29 Phytophthora infestans T30-4
A Toxoplasma gondii RH
31 Rhodosorus marinus
A Galdieria sulphuraria
20 Nasonia vitripennis
F Eutreptiella gymnastica
E Trypanosoma cruzi strain CL Brener
A Naegleria gruberi strain NEG-M
C Eutreptiella gymnastica
13 Platyophrya macrostoma
13 Leishmania infantum JPCM5
7 Mayorella sp. JJP-2003
I Acanthamoeba castellanii str. Neff
22 Cavenderia fasciculata
I Dictyostelium discoideum AX4
I Gloeochaete wittrockiana
15 prasinophyte sp. RCC997
22 Spizellomyces punctatus DAOM BR117
22 Capsaspora owczarzaki ATCC 30864
22 Monosiga brevicollis
22 Nasonia vitripennis
G Dictyostelium discoideum AX4
32 Hyaloperonospora parasitica
E Capsaspora owczarzaki ATCC 30864
1 Thecamonas trahens ATCC 50062
1 Monosiga brevicollis
33 Synchytrium microbalum
1 Capsaspora owczarzaki ATCC 30864
1 Nasonia vitripennis
1 Naegleria gruberi strain NEG-M
1 Lotharella globosa
1 Trichosphaerium sp. ATCC 40318
1 Acanthamoeba castellanii
1 Palpitomonas bilix
1 Hyaloperonospora parasitica
1 Leishmania infantum JPCM5
1 Fonticula alba
1 Dictyostelium discoideum AX4
1 Quercus suber
1 Neurospora crassa OR74A
1 prasinophyte sp. RCC997
1 Gloeochaete wittrockiana
10 Capsaspora owczarzaki ATCC 30864
10 Monosiga brevicollis
10 Takifugu rubripes
7 Nasonia vitripennis
G Necator americanus
7 Rhodamnia argentea
32 Biomphalaria glabrata
7 Monosiga brevicollis
11 Amphimedon queenslandica
U Colius striatus
13 Paramormyrops kingsleyae
I Chlamydotis macqueenii
14 Gymnodraco acuticeps
29 Gymnodraco acuticeps
G Capsaspora owczarzaki ATCC 30864
15 Capsaspora owczarzaki ATCC 30864
35 Takifugu rubripes
15 Monosiga brevicollis
15 Nasonia vitripennis
9 Nasonia vitripennis
16 Takifugu rubripes
N Monosiga brevicollis
28 Salpingoeca rosetta
O Monosiga brevicollis
28 Xenopus tropicalis
C Strongylocentrotus purpuratus
G Monosiga brevicollis
H Monosiga brevicollis
D Monosiga brevicollis
E Monosiga brevicollis
L Monosiga brevicollis
M Monosiga brevicollis
I Monosiga brevicollis
J Monosiga brevicollis
C Monosiga brevicollis
A Monosiga brevicollis
K Monosiga brevicollis
P Monosiga brevicollis
F Monosiga brevicollis
B Strongylocentrotus purpuratus
O Hydra vulgaris
21 Nasonia vitripennis
3 Nasonia vitripennis
H Acyrthosiphon pisum
J Scyliorhinus canicula
L Scyliorhinus canicula
N Bos taurus
M Erpetoichthys calabaricus

E

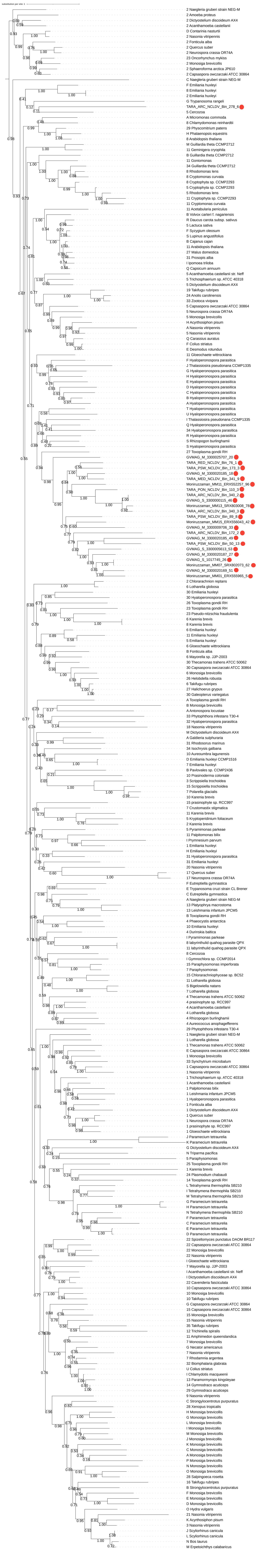

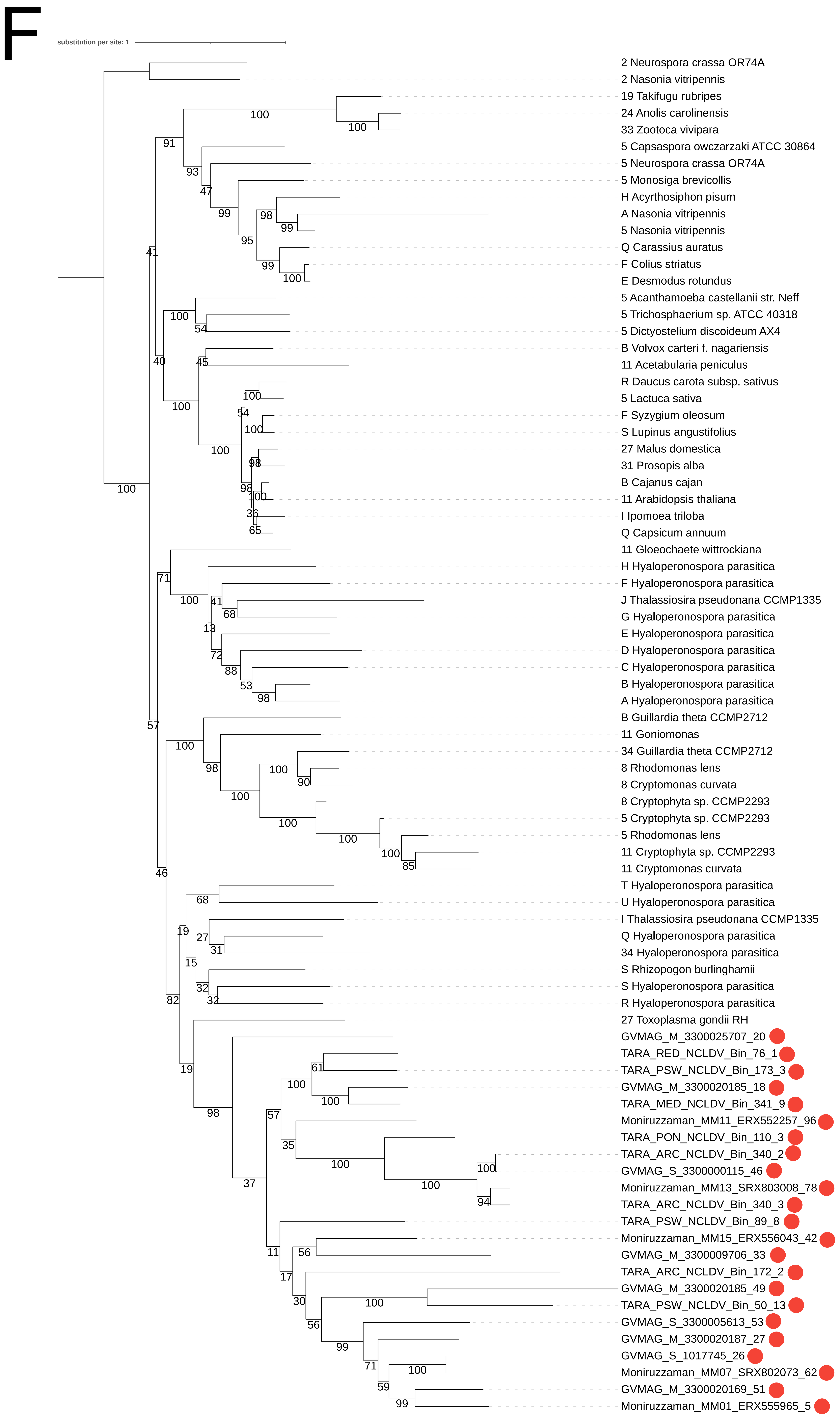
F
substitution per site: 1
2 Neurospora crassa OR74A
2 Nasonia vitripennis
19 Takifugu rubripes
24 Anolis carolinensis
33 Zootoca vivipara
5 Capsaspora owczarzaki ATCC 30864
5 Neurospora crassa OR74A
5 Monosiga brevicollis
H Acyrthosiphon pisum
A Nasonia vitripennis
5 Nasonia vitripennis
Q Carassius auratus
F Colius striatus
E Desmodus rotundus
5 Acanthamoeba castellanii str. Neff
5 Trichosphaerium sp. ATCC 40318
5 Dictyostelium discoideum AX4
B Volvox carteri f. nagariensis
11 Acetabularia peniculus
R Daucus carota subsp. sativus
5 Lactuca sativa
F Syzygium oleosum
S Lupinus angustifolius
27 Malus domestica
31 Prosopis alba
B Cajanus cajan
11 Arabidopsis thaliana
I Ipomoea triloba
Q Capsicum annuum
11 Gloeochaete wittrockiana
H Hyaloperonospora parasitica
F Hyaloperonospora parasitica
J Thalassiosira pseudonana CCMP1335
G Hyaloperonospora parasitica
E Hyaloperonospora parasitica
D Hyaloperonospora parasitica
C Hyaloperonospora parasitica
B Hyaloperonospora parasitica
A Hyaloperonospora parasitica
B Guillardia theta CCMP2712
11 Goniomonas
34 Guillardia theta CCMP2712
8 Rhodomonas lens
8 Cryptomonas curvata
8 Cryptophyta sp. CCMP2293
5 Cryptophyta sp. CCMP2293
5 Rhodomonas lens
11 Cryptophyta sp. CCMP2293
11 Cryptomonas curvata
T Hyaloperonospora parasitica
U Hyaloperonospora parasitica
I Thalassiosira pseudonana CCMP1335
Q Hyaloperonospora parasitica
34 Hyaloperonospora parasitica
S Rhizopogon burlinghamii
S Hyaloperonospora parasitica
R Hyaloperonospora parasitica
27 Toxoplasma gondii RH
GVMAG_M_3300025707_20
TARA_RED_NCLDV_Bin_76_1
TARA_PSW_NCLDV_Bin_173_3
GVMAG_M_3300020185_18
TARA_MED_NCLDV_Bin_341_9
Moniruzzaman_MM11_ERX552257_96
TARA_PON_NCLDV_Bin_110_3
TARA_ARC_NCLDV_Bin_340_2
GVMAG_S_3300000115_46
Moniruzzaman_MM13_SRX803008_78
TARA_ARC_NCLDV_Bin_340_3
TARA_PSW_NCLDV_Bin_89_8
Moniruzzaman_MM15_ERX556043_42
GVMAG_M_3300009706_33
TARA_ARC_NCLDV_Bin_172_2
GVMAG_M_3300020185_49
TARA_PSW_NCLDV_Bin_50_13
GVMAG_S_3300005613_53
GVMAG_M_3300020187_27
GVMAG_S_1017745_26
Moniruzzaman_MM07_SRX802073_62
GVMAG_M_3300020169_51
Moniruzzaman_MM01_ERX555965_5

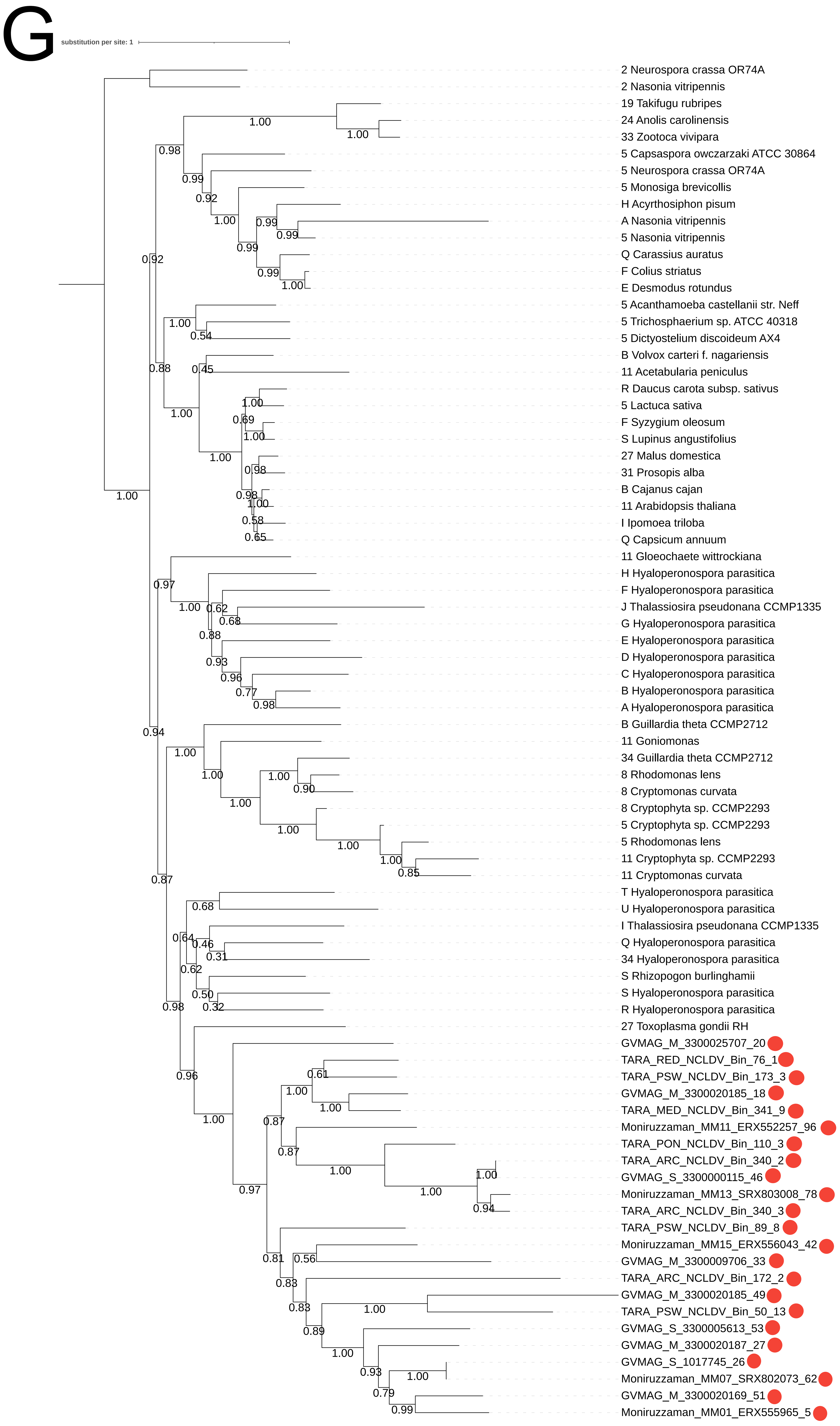

G
substitution per site: 1
2 Neurospora crassa OR74A
2 Nasonia vitripennis
19 Takifugu rubripes
24 Anolis carolinensis
33 Zootoca vivipara
5 Capsaspora owczarzaki ATCC 30864
5 Neurospora crassa OR74A
5 Monosiga brevicollis
H Acyrthosiphon pisum
A Nasonia vitripennis
5 Nasonia vitripennis
Q Carassius auratus
F Colius striatus
E Desmodus rotundus
5 Acanthamoeba castellanii str. Neff
5 Trichosphaerium sp. ATCC 40318
5 Dictyostelium discoideum AX4
B Volvox carteri f. nagariensis
11 Acetabularia peniculus
R Daucus carota subsp. sativus
5 Lactuca sativa
F Syzygium oleosum
S Lupinus angustifolius
27 Malus domestica
31 Prosopis alba
B Cajanus cajan
11 Arabidopsis thaliana
I Ipomoea triloba
Q Capsicum annuum
11 Gloeochaete wittrockiana
H Hyaloperonospora parasitica
F Hyaloperonospora parasitica
J Thalassiosira pseudonana CCMP1335
G Hyaloperonospora parasitica
E Hyaloperonospora parasitica
D Hyaloperonospora parasitica
C Hyaloperonospora parasitica
B Hyaloperonospora parasitica
A Hyaloperonospora parasitica
B Guillardia theta CCMP2712
11 Goniomonas
34 Guillardia theta CCMP2712
8 Rhodomonas lens
8 Cryptomonas curvata
8 Cryptophyta sp. CCMP2293
5 Cryptophyta sp. CCMP2293
5 Rhodomonas lens
11 Cryptophyta sp. CCMP2293
11 Cryptomonas curvata
T Hyaloperonospora parasitica
U Hyaloperonospora parasitica
I Thalassiosira pseudonana CCMP1335
Q Hyaloperonospora parasitica
34 Hyaloperonospora parasitica
S Rhizopogon burlinghamii
S Hyaloperonospora parasitica
R Hyaloperonospora parasitica
27 Toxoplasma gondii RH
GVMAG_M_3300025707_20
TARA_RED_NCLDV_Bin_76_1
TARA_PSW_NCLDV_Bin_173_3
GVMAG_M_3300020185_18
TARA_MED_NCLDV_Bin_341_9
Moniruzzaman_MM11_ERX552257_96
TARA_PON_NCLDV_Bin_110_3
TARA_ARC_NCLDV_Bin_340_2
GVMAG_S_3300000115_46
Moniruzzaman_MM13_SRX803008_78
TARA_ARC_NCLDV_Bin_340_3
TARA_PSW_NCLDV_Bin_89_8
Moniruzzaman_MM15_ERX556043_42
GVMAG_M_3300009706_33
TARA_ARC_NCLDV_Bin_172_2
GVMAG_M_3300020185_49
TARA_PSW_NCLDV_Bin_50_13
GVMAG_S_3300005613_53
GVMAG_M_3300020187_27
GVMAG_S_1017745_26
Moniruzzaman_MM07_SRX802073_62
GVMAG_M_3300020169_51
Moniruzzaman_MM01_ERX555965_5

H

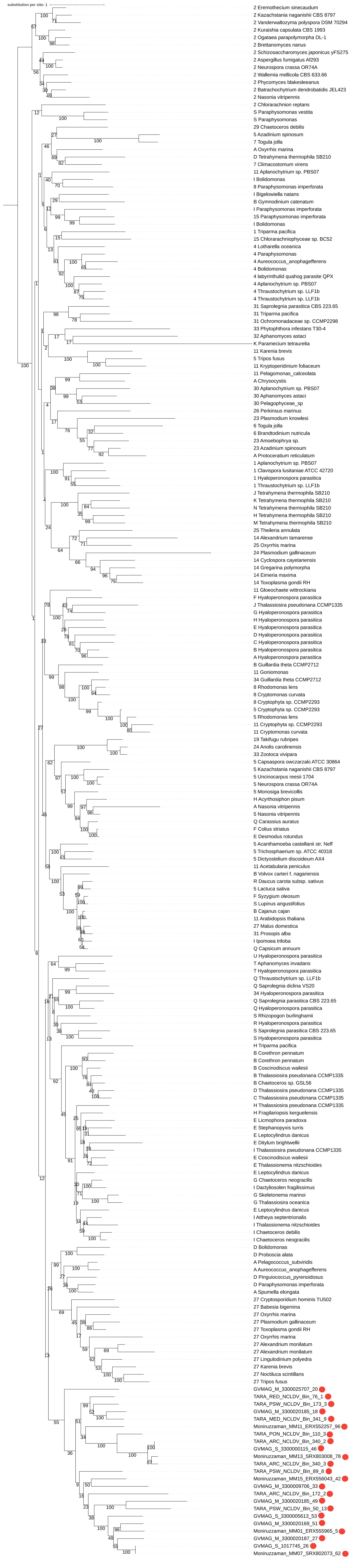
substitution per site: 1
2 Eremothecium sinecaudum
2 Kazachstania naganishii CBS 8797
2 Vanderwaltozyma polyspora DSM 70294
2 Kuraishia capsulata CBS 1993
2 Ogataea parapolymorpha DL-1
2 Brettanomyces nanus
2 Schizosaccharomyces japonicus yFS275
2 Aspergillus fumigatus Af293
2 Neurospora crassa OR74A
2 Wallemia mellicola CBS 633.66
2 Phycomyces blakesleeanus
2 Batrachochytrium dendrobatidis JEL423
2 Nasonia vitripennis
2 Chlorarachnion reptans
S Paraphysomonas vestita
S Paraphysomonas
29 Chaetoceros debilis
5 Azadinium spinosum
7 Togula jolla
A Oxyrrhis marina
D Tetrahymena thermophila SB210
7 Climacostomum virens
11 Aplanochytrium sp. PBS07
I Bolidomonas
8 Paraphysomonas imperforata
I Bigelowiella natans
B Gymnodinium catenatum
I Paraphysomonas imperforata
15 Paraphysomonas imperforata
I Bolidomonas
1 Triparma pacifica
15 Chlorarachniophyceae sp. BC52
4 Lotharella oceanica
4 Paraphysomonas
4 Aureococcus_anophagefferens
4 Bolidomonas
4 labyrinthulid quahog parasite QPX
4 Aplanochytrium sp. PBS07
4 Thraustochytrium sp. LLF1b
4 Thraustochytrium sp. LLF1b
31 Saprolegnia parasitica CBS 223.65
31 Triparma pacifica
31 Ochromonadaceae sp. CCMP2298
33 Phytophthora infestans T30-4
32 Aphanomyces astaci
K Paramecium tetraurelia
11 Karenia brevis
5 Tripos fusus
11 Kryptoperidinium foliaceum
11 Pelagomonas_calceolata
A Chrysocystis
30 Aplanochytrium sp. PBS07
30 Aphanomyces astaci
30 Pelagophyceae_sp
26 Perkinsus marinus
23 Plasmodium knowlesi
6 Togula jolla
6 Brandtodinium nutricula
23 Amoebophrya sp.
23 Azadinium spinosum
A Protoceratium reticulatum
1 Aplanochytrium sp. PBS07
1 Clavispora lusitaniae ATCC 42720
1 Hyaloperonospora parasitica
1 Thraustochytrium sp. LLF1b
J Tetrahymena thermophila SB210
K Tetrahymena thermophila SB210
N Tetrahymena thermophila SB210
H Tetrahymena thermophila SB210
M Tetrahymena thermophila SB210
25 Theileria annulata
14 Alexandrium tamarense
25 Oxyrrhis marina
24 Plasmodium gallinaceum
14 Cyclospora cayetanensis
14 Gregarina polymorpha
14 Eimeria maxima
14 Toxoplasma gondii RH
11 Gloeochaete wittrockiana
F Hyaloperonospora parasitica
J Thalassiosira pseudonana CCMP1335
G Hyaloperonospora parasitica
H Hyaloperonospora parasitica
E Hyaloperonospora parasitica
D Hyaloperonospora parasitica
C Hyaloperonospora parasitica
B Hyaloperonospora parasitica
A Hyaloperonospora parasitica
B Guillardia theta CCMP2712
11 Goniomonas
34 Guillardia theta CCMP2712
8 Rhodomonas lens
8 Cryptomonas curvata
8 Cryptophyta sp. CCMP2293
5 Cryptophyta sp. CCMP2293
5 Rhodomonas lens
11 Cryptophyta sp. CCMP2293
11 Cryptomonas curvata
19 Takifugu rubripes
24 Anolis carolinensis
33 Zootoca vivipara
5 Capsaspora owczarzaki ATCC 30864
5 Kazachstania naganishii CBS 8797
5 Uncinocarpus reesii 1704
5 Neurospora crassa OR74A
5 Monosiga brevicollis
H Acyrthosiphon pisum
A Nasonia vitripennis
5 Nasonia vitripennis
Q Carassius auratus
F Colius striatus
E Desmodus rotundus
5 Acanthamoeba castellanii str. Neff
5 Trichosphaerium sp. ATCC 40318
5 Dictyostelium discoideum AX4
11 Acetabularia peniculus
B Volvox carteri f. nagariensis
R Daucus carota subsp. sativus
5 Lactuca sativa
F Syzygium oleosum
S Lupinus angustifolius
B Cajanus cajan
11 Arabidopsis thaliana
27 Malus domestica
31 Prosopis alba
I Ipomoea triloba
Q Capsicum annuum
U Hyaloperonospora parasitica
T Aphanomyces invadans
S Hyaloperonospora parasitica
Q Thraustochytrium sp. LLF1b
Q Saprolegnia diclina VS20
34 Hyaloperonospora parasitica
Q Saprolegnia parasitica CBS 223.65
Q Hyaloperonospora parasitica
S Rhizopogon burlinghamii
R Hyaloperonospora parasitica
S Saprolegnia parasitica CBS 223.65
S Hyaloperonospora parasitica
H Triparma pacifica
B Corethron pennatum
B Corethron pennatum
B Coscinodiscus wailesii
B Thalassiosira pseudonana CCMP1335
B Chaetoceros sp. GSL56
D Thalassiosira pseudonana CCMP1335
C Thalassiosira pseudonana CCMP1335
H Thalassiosira pseudonana CCMP1335
H Fragilariopsis kerguelensis
E Licmophora paradoxa
E Stephanopyxis turris
E Leptocylindrus danicus
E Ditylum brightwellii
I Thalassiosira pseudonana CCMP1335
E Coscinodiscus wailesii
E Thalassionema nitzschioides
E Leptocylindrus danicus
G Chaetoceros neogracilis
I Dactyliosolen fragilissimus
G Skeletonema marinoi
G Thalassiosira oceanica
E Leptocylindrus danicus
I Attheya septentrionalis
I Thalassionema nitzschioides
I Chaetoceros debilis
I Chaetoceros neogracilis
D Bolidomonas
D Proboscia alata
A Pelagococcus_subviridis
A Aureococcus_anophagefferens
D Pinguiococcus_pyrenoidosus
D Paraphysomonas imperforata
A Spumella elongata
27 Cryptosporidium hominis TU502
27 Babesia bigemina
27 Oxyrrhis marina
27 Plasmodium gallinaceum
27 Toxoplasma gondii RH
27 Oxyrrhis marina
27 Alexandrium monilatum
27 Alexandrium monilatum
27 Lingulodinium polyedra
27 Karenia brevis
27 Noctiluca scintillans
27 Tripos fusus
GVMAG_M_3300025707_20
TARA_RED_NCLDV_Bin_76_1
TARA_PSW_NCLDV_Bin_173_3
GVMAG_M_3300020185_18
TARA_MED_NCLDV_Bin_341_9
Moniruzzaman_MM11_ERX552257_96
TARA_PON_NCLDV_Bin_110_3
TARA_ARC_NCLDV_Bin_340_2
GVMAG_S_3300000115_46
Moniruzzaman_MM13_SRX803008_78
TARA_ARC_NCLDV_Bin_340_3
TARA_PSW_NCLDV_Bin_89_8
Moniruzzaman_MM15_ERX556043_42
GVMAG_M_3300009706_33
TARA_ARC_NCLDV_Bin_172_2
GVMAG_M_3300020185_49
TARA_PSW_NCLDV_Bin_50_13
GVMAG_S_3300005613_53
GVMAG_M_3300020169_51
Moniruzzaman_MM01_ERX555965_5
GVMAG_M_3300020187_27
GVMAG_S_1017745_26
Moniruzzaman_MM07_SRX802073_62

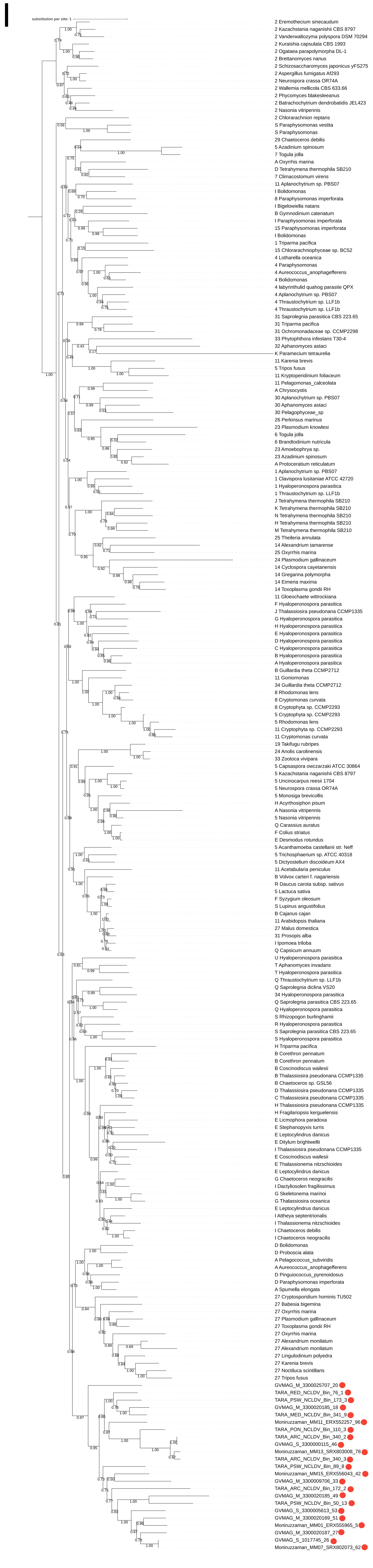
substitution per site: 1
2 Eremothecium sinecaudum
2 Kazachstania naganishii CBS 8797
2 Vanderwaltozyma polyspora DSM 70294
2 Kuraishia capsulata CBS 1993
2 Ogataea parapolymorpha DL-1
2 Brettanomyces nanus
2 Schizosaccharomyces japonicus yFS275
2 Aspergillus fumigatus Af293
2 Neurospora crassa OR74A
2 Wallemia mellicola CBS 633.66
2 Phycomyces blakesleeanus
2 Batrachochytrium dendrobatidis JEL423
2 Nasonia vitripennis
2 Chlorarachnion reptans
S Paraphysomonas vestita
S Paraphysomonas
29 Chaetoceros debilis
5 Azadinium spinosum
7 Togula jolla
A Oxyrrhis marina
D Tetrahymena thermophila SB210
7 Climacostomum virens
11 Aplanochytrium sp. PBS07
I Bolidomonas
8 Paraphysomonas imperforata
I Bigelowiella natans
B Gymnodinium catenatum
I Paraphysomonas imperforata
15 Paraphysomonas imperforata
I Bolidomonas
1 Triparma pacifica
15 Chlorarachniophyceae sp. BC52
4 Lotharella oceanica
4 Paraphysomonas
4 Aureococcus_anophagefferens
4 Bolidomonas
4 labyrinthulid quahog parasite QPX
4 Aplanochytrium sp. PBS07
4 Thraustochytrium sp. LLF1b
4 Thraustochytrium sp. LLF1b
31 Saprolegnia parasitica CBS 223.65
31 Triparma pacifica
31 Ochromonadaceae sp. CCMP2298
33 Phytophthora infestans T30-4
32 Aphanomyces astaci
K Paramecium tetraurelia
11 Karenia brevis
5 Tripos fusus
11 Kryptoperidinium foliaceum
11 Pelagomonas_calceolata
A Chrysocystis
30 Aplanochytrium sp. PBS07
30 Aphanomyces astaci
30 Pelagophyceae_sp
26 Perkinsus marinus
23 Plasmodium knowlesi
6 Togula jolla
6 Brandtodinium nutricula
23 Amoebophrya sp.
23 Azadinium spinosum
A Protoceratium reticulatum
1 Aplanochytrium sp. PBS07
1 Clavispora lusitaniae ATCC 42720
1 Hyaloperonospora parasitica
1 Thraustochytrium sp. LLF1b
J Tetrahymena thermophila SB210
K Tetrahymena thermophila SB210
N Tetrahymena thermophila SB210
H Tetrahymena thermophila SB210
M Tetrahymena thermophila SB210
25 Theileria annulata
14 Alexandrium tamarense
25 Oxyrrhis marina
24 Plasmodium gallinaceum
14 Cyclospora cayetanensis
14 Gregarina polymorpha
14 Eimeria maxima
14 Toxoplasma gondii RH
11 Gloeochaete wittrockiana
F Hyaloperonospora parasitica
J Thalassiosira pseudonana CCMP1335
G Hyaloperonospora parasitica
H Hyaloperonospora parasitica
E Hyaloperonospora parasitica
D Hyaloperonospora parasitica
C Hyaloperonospora parasitica
B Hyaloperonospora parasitica
A Hyaloperonospora parasitica
B Guillardia theta CCMP2712
11 Goniomonas
34 Guillardia theta CCMP2712
8 Rhodomonas lens
8 Cryptomonas curvata
8 Cryptophyta sp. CCMP2293
5 Cryptophyta sp. CCMP2293
5 Rhodomonas lens
11 Cryptophyta sp. CCMP2293
11 Cryptomonas curvata
19 Takifugu rubripes
24 Anolis carolinensis
33 Zootoca vivipara
5 Capsaspora owczarzaki ATCC 30864
5 Kazachstania naganishii CBS 8797
5 Uncinocarpus reesii 1704
5 Neurospora crassa OR74A
5 Monosiga brevicollis
H Acyrthosiphon pisum
A Nasonia vitripennis
5 Nasonia vitripennis
Q Carassius auratus
F Colius striatus
E Desmodus rotundus
5 Acanthamoeba castellanii str. Neff
5 Trichosphaerium sp. ATCC 40318
5 Dictyostelium discoideum AX4
11 Acetabularia peniculus
B Volvox carteri f. nagariensis
R Daucus carota subsp. sativus
5 Lactuca sativa
F Syzygium oleosum
S Lupinus angustifolius
B Cajanus cajan
11 Arabidopsis thaliana
27 Malus domestica
31 Prosopis alba
I Ipomoea triloba
Q Capsicum annuum
U Hyaloperonospora parasitica
T Aphanomyces invadans
S Hyaloperonospora parasitica
Q Thraustochytrium sp. LLF1b
Q Saprolegnia diclina VS20
34 Hyaloperonospora parasitica
Q Saprolegnia parasitica CBS 223.65
Q Hyaloperonospora parasitica
S Rhizopogon burlinghamii
R Hyaloperonospora parasitica
S Saprolegnia parasitica CBS 223.65
S Hyaloperonospora parasitica
H Triparma pacifica
B Corethron pennatum
B Corethron pennatum
B Coscinodiscus wailesii
B Thalassiosira pseudonana CCMP1335
B Chaetoceros sp. GSL56
D Thalassiosira pseudonana CCMP1335
C Thalassiosira pseudonana CCMP1335
H Thalassiosira pseudonana CCMP1335
H Fragilariopsis kerguelensis
E Licmophora paradoxa
E Stephanopyxis turris
E Leptocylindrus danicus
E Ditylum brightwellii
I Thalassiosira pseudonana CCMP1335
E Coscinodiscus wailesii
E Thalassionema nitzschioides
E Leptocylindrus danicus
G Chaetoceros neogracilis
I Dactyliosolen fragilissimus
G Skeletonema marinoi
G Thalassiosira oceanica
E Leptocylindrus danicus
I Attheya septentrionalis
I Thalassionema nitzschioides
I Chaetoceros debilis
I Chaetoceros neogracilis
D Bolidomonas
D Proboscia alata
A Pelagococcus_subviridis
A Aureococcus_anophagefferens
D Pinguiococcus_pyrenoidosus
D Paraphysomonas imperforata
A Spumella elongata
27 Cryptosporidium hominis TU502
27 Babesia bigemina
27 Oxyrrhis marina
27 Plasmodium gallinaceum
27 Toxoplasma gondii RH
27 Oxyrrhis marina
27 Alexandrium monilatum
27 Alexandrium monilatum
27 Lingulodinium polyedra
27 Karenia brevis
27 Noctiluca scintillans
27 Tripos fusus
GVMAG_M_3300025707_20
TARA_RED_NCLDV_Bin_76_1
TARA_PSW_NCLDV_Bin_173_3
GVMAG_M_3300020185_18
TARA_MED_NCLDV_Bin_341_9
Moniruzzaman_MM11_ERX552257_96
TARA_PON_NCLDV_Bin_110_3
TARA_ARC_NCLDV_Bin_340_2
GVMAG_S_3300000115_46
Moniruzzaman_MM13_SRX803008_78
TARA_ARC_NCLDV_Bin_340_3
TARA_PSW_NCLDV_Bin_89_8
Moniruzzaman_MM15_ERX556043_42
GVMAG_M_3300009706_33
TARA_ARC_NCLDV_Bin_172_2
GVMAG_M_3300020185_49
TARA_PSW_NCLDV_Bin_50_13
GVMAG_S_3300005613_53
GVMAG_M_3300020169_51
Moniruzzaman_MM01_ERX555965_5
GVMAG_M_3300020187_27
GVMAG_S_1017745_26
Moniruzzaman_MM07_SRX802073_62
